# Printed Conformal and Transparent Magnetoresistive Sensors for Seamless Integration and Environment-Resilient Touchless Interaction

**DOI:** 10.1021/acsnano.5c07664

**Published:** 2025-06-05

**Authors:** Rui Xu, Eduardo Sergio Oliveros Mata, Fei Cheng, Oleksandr V. Pylypovskyi, Qihao Zhang, Proloy Taran Das, Yevhen Zabila, Olha Bezsmertna, Jun Yang, Xiaotao Wang, Sebastian Lehmann, Lin Guo, René Hübner, Fabian Ganss, Ran He, Rico Illing, Kornelius Nielsch, Denys Makarov

**Affiliations:** † Institute of Ion Beam Physics and Materials Research, 28414 Helmholtz-Zentrum Dresden-Rossendorf e.V., Bautzner Landstrasse 400, Dresden 01328, Germany; ‡ Xi’an Rare Metal Materials Institute Co., Ltd., Xi’an 710016, P. R. China; § Institute for Metallic Materials, 28394 Leibniz Institute for Solid State and Materials Research, Dresden 01069, Germany; ∥ Institute of Materials Science, Technische Universität Dresden, Dresden 01062, Germany

**Keywords:** magnetoresistive sensor, printable magnetoelectronics, transparent electronics, flexible electronics, magnetic nanowire

## Abstract

Combination of conformability and transparency is crucial for realizing the full capabilities of printed magnetoresistive sensors in cutting-edge technologies designed to blend into their surroundings and applications. However, achieving this poses a critical challenge due to conflicting requirements: magnetic nanowires optimized for deformability exhibit a tendency to cluster, thus compromising transparency. To balance this trade-off, we leverage magnetic fields to manipulate nanowires, simultaneously initiating alignment and pinning effects. These together ensure a uniform and anisotropic distribution across extensive areas, enhancing the sensor transparency (about 85%). Further, we harness the clustering tendency, repurposing it to create local entanglements that enhance mechanical durability against both bending (with a curvature radius of about 110 μm) and stretching (with 80% tensile strain) and result in stable performance during 10,000 magnetization cycles. With the anisotropic design, the printed sensors achieve high out-of-plane sensitivity, distinguishing them from traditional film-based counterparts with a predominant in-plane response. These sensors do not require physical contact during operation, fostering hygienic and safer interaction. Their robust performance under environmental interference (e.g., dust, liquid, and moisture) makes them versatile for real-world use. The above innovations position our sensor as an important driver across numerous emerging applications, e.g., touchless interactive transparent displays and integrated multifunctional windows.

## Graphical abstract

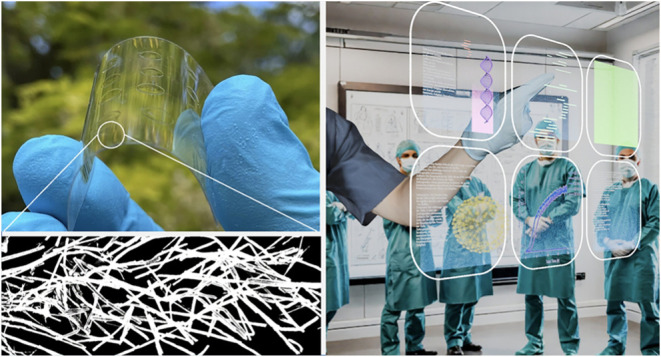

## Introduction

Our world is rapidly evolving into a tightly interconnected entity, driving the demand for a diverse array of functional devices capable of synergistic collaboration to enable groundbreaking functionalities.^1–4^ The cornerstone of this transformation lies in integrating these devices into cohesive systems without compromising their main functionalities, requiring them to possess a variety of unique properties. Among these, mechanical conformability and visual transparency are gaining increasing attention, as they facilitate the seamless integration of these devices into environments and applications that need to conform to nonplanar or biodynamic surfaces^5–8^ or to maintain visual information.^9–11^ To this end, a wide range of flexible and transparent electronic elements have been developed, including electrodes,^12,13^ transistors,^14,15^ memristors,^16^ solar cells,^17^ supercapacitors,^18^ heaters,^19^ diverse sensors,^20^ etc. Among these innovations, magnetoresistive sensors stand out as a vital member of the electronic component family thanks to their capability of converting magnetic stimuli to electrical signals.^21–23^ It makes them indispensable for applications requiring precise information exchange and rapid interaction, e.g., in medical equipment, space technology, automobile industry, environmental monitoring, and human–machine interaction. Despite these advancements, the absence of a conformal and transparent magnetoresistive sensor remains a critical limitation, posing significant barriers to their adoption into emerging applications.

Conventional fabrication methods, typically based on semiconductor-processing techniques, can fabricate specific patterns with wavy configuration and ultralow material coverage to gain conformability and transparency.^24–26^ However, their reliance on advanced equipment and subtractive manufacturing complicates the fabrication process, reduces material efficiency, and drives up production costs. In contrast, ink-printing techniques offer a low-cost, environment-friendly approach for mass production of magnetoresistive sensors.^27–29^ To date, printed bendable magnetoresistive sensors have been reported.^30–32^ Stretchability was successfully introduced by prestraining the hosting substrates.^33^ However, the resulting wrinkled structures are not appropriate for applications demanding smooth or conformal surfaces. Notably, all of the reported magnetoresistive sensors lack transparency constrained by the strong light absorption of magnetic fillers, evidenced by the black color of both magnetic nanowire- and nanoparticle-based inks in refs 34,35 , and this work (Figure 1a).^34,35^ Incorporating nanowire-shaped fillers to create mesh-like networks holds promise for improving both transparency and conformability.^36–39^ Although many methods have been developed to synthesize magnetic nanowires (e.g., through semiconductor-processing techniques,^40^ chemical reactions,^41–44^ or template-aided electrodepostion^45,46^ ), the development of conformal and transparent magnetoresistive sensors remains challenging.

**Figure 1:**
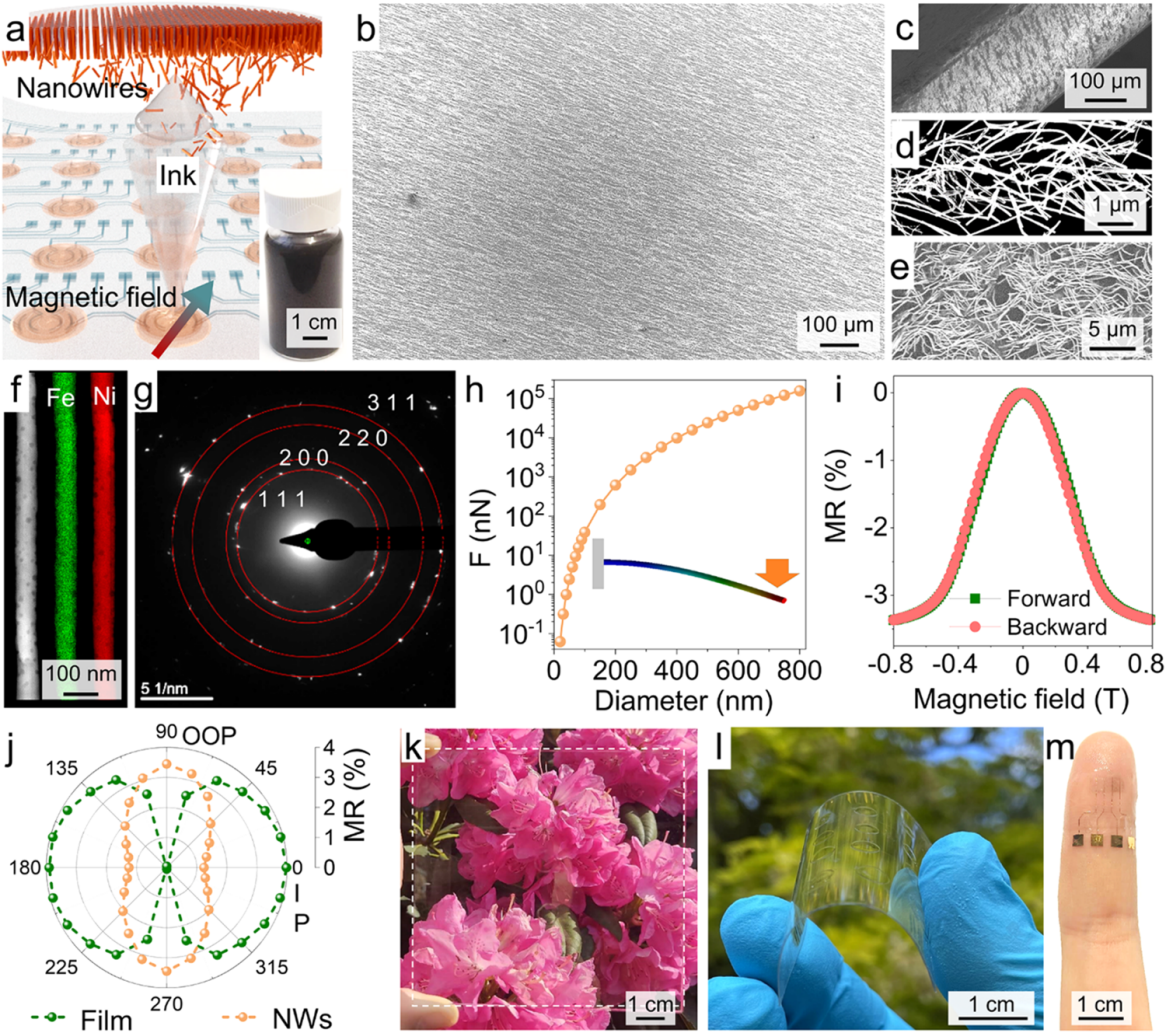
Printed conformal and transparent magnetoresistive sensors. (a) Schematic illustration of the printing process, guided by magnetic fields. NiFe nanowires, synthesized using a template-mediated electrodeposition technique, are dispersed in isopropanol to create functional inks, as shown in the inset. (b) Printed nanowires over a large area. (c) Printed network, bent with a curvature radius of about 110 μm. (d) Zoom-in view of the printed nanowire network, characterized with mechanical entanglement. (e) Printed nanowires, after the release of prestrain, exhibiting arc- or kink-shaped structures along their length. (f) Energy-dispersive X-ray spectroscopy (EDXS) of magnetoresistive nanowires made of NiFe alloy. (g) Selected-area electron diffraction pattern of a single NiFe nanowire obtained in the transmission electron microscopy (TEM). (h) Numerically calculated mechanical force, required to bend diameter-different nanowires to a specific deformation. Inset: schematic illustration of a nanowire under an external force (represented by an arrow). (i) Room-temperature magnetoresistance (MR) response of the printed sensor, with magnetic fields applied along the nanowire alignment direction. Measurements are taken by sweeping magnetic fields in both the forward and reverse directions. (j) Angle-dependent magnetoresistance (MR) response of the nanowire-based sensor compared to its film-based counterpart, with magnetic fields gradually transitioning from the in-plane alignment-parallel direction to the out-of-plane (OOP) perpendicular direction. (k) Nanowire networks printed on glass, exhibiting high transparency. (l) Transparent networks printed on a flexible polyethylene terephthalate (PET) substrate in a bent state. (m) Printed conformal and transparent magnetoresistive sensor, mounted onto a finger.

The technical difficulties lie in an intricate combination of conformability (bendability and stretchability) and transparency. Combining membrane filtration and transfer printing techniques has enabled bendable yet nonstretchable magnetoresistive networks.^47^ Incorporating highly conductive nanowires promotes electrical percolation for low-concentration inks and thus can improve transparency, but it will compromise the magnetoresistive response due to shunting currents.^48,49^ Alternatively, the directional alignment of functional nanowires has been explored to enhance both transparency and device performance. Magnetic wires with sub μm diameters can be aligned along magnetic fields, behaving as magnetic dipoles.^50,51^ However, achieving a uniform network over large areas is not a trivial matter. Particularly, the higher Young’s modulus of magnetic materials (Co, Ni, Fe), more than twice that of Au and Ag, limits their deformability. This results in weak physical contact between printed magnetic nanowires, making the resulting electrical pathways prone to disruption under continuous bending, and enabling stretchable sensors. Reducing the nanowire diameter could mitigate this problem. However, this reduction increases the surface-to-volume ratio, intensifying surficial interaction and promoting nanowire aggregation during the printing process,^52,53^ as evidenced in Supporting Video S1. Moreover, bent nanowires, caused by enhanced deformability, exhibit complex magnetization, which might trigger unexpected interactions with neighboring nanowires and further exacerbate clustering issues.^54,55^ Therefore, to realize conformal and transparent magnetoresistive sensors, it is imperative to devise a method to balance multiple conflicting demands.

In this work, we realize locally entangled and large-area aligned magnetoresistive networks by leveraging magnetic fields to guide the arrangement of NiFe nanowires. Experiments and simulations reveal that the guiding fields must be oriented at certain angles relative to the printed inks, with the parallel field components extending percolation paths and the perpendicular components mitigating unfavorable clustering. Mechanical entanglement of the nanowires leads to robust electrical pathways and consistent sensing performance against bending and stretching. Directional alignment effectively increases optical transparency (about 85%) while maintaining electrical percolation at the alignment direction. Thanks to the anisotropic configuration, the printed sensors exhibit exceptional out-of-plane sensitivity, ensuring reliable operation in real-world scenarios, where magnetic sources may approach from any direction. These unique properties allow the sensors to seamlessly integrate into diverse systems to perform synergistic collaboration, as proved by touchless interactive transparent displays and smart windows with a combination of sensing, transparency-tuning, and thermoelectric conversion capabilities. By leveraging the touchless interactive mode, these intelligent systems hold great promise for applications in environments with stringent demands for interactive safety and hygiene. Even when exposed to surface contaminants (e.g., water and stains), the magnetoresistive sensors maintain operational immunity, offering an environmentally resilient solution for touchless interaction in harsh conditions.

## Results

### Printed Magnetoresistive Networks with Mechanical Conformability and Visual Transparency

Figure 1a presents a schematic illustration of the sensor fabrication process, which utilizes magnetic fields to guide the printing process, giving rise to mechanically stable and directionally aligned networks (Figure 1b,c) with local entanglement (Figure 1d) and wavy patterns (Figure 1e). To promote mechanical conformability, visual transparency, and magnetoresistance response, we meticulously tailor both the fillers and their networks. Traditional semiconductor-processing techniques offer reliable approaches to fabricate high-magnetoresistance nanowires;^40,56^ however, they are both costly and time-intensive. Chemical methods (e.g., hydrothermal processing^41,42^ or chemical reduction^34,43,44^ ) are suited for mass production, but the obtained nanowires often suffer from low quality and high impurity, adversely impacting magnetoresistive response. In this context, we exploit template-aided electrodeposition methods for large-scale synthesis of magnetoresistive nanowires (Figure S1).^45,46,57^ Thanks to the geometrical tunability of nanoporous aluminum oxide templates,^58–60^ the nanowire diameter can be readily adjusted from hundreds down to tens of nanometers (Figure S2), with lengths extending beyond 100 μm (Figure S3). As shown in Figure 1f, the NiFe nanowire demonstrates a smoother surface relative to its counterparts synthesized via hydrothermal methods,^34,41^ which is desirable for magnetoresistance enhancement by suppressing electron surface scattering. The elements Ni and Fe distribute throughout the nanowire without noticeable separation, which is typically a characteristic of alloy formation. Figure 1g presents a selected-area electron diffraction pattern of a single NiFe nanowire, obtained via transmission electron microscope (TEM). The diffraction rings correspond to the {111}, {200}, {220}, and {311} lattice planes, aligning with the X-ray diffraction (XRD) pattern in Figure S4. These results confirm the successful formation of a face-centered cubic (FCC) NiFe alloy structure.^61^ Considering the high Young’s modulus of magnetic nanowires, their diameters are reduced to enhance mechanical deformability. As the nanowire diameter approaches the nanometer scale, these structures become more prone to mechanical stress (Figure 1h). The simulation result is consistent with experimental observation showing that significant deformation detected in 65 nm diameter nanowires is barely observed in nanowire networks with diameters of 150 and 350 nm (Figures 1c–e and S5). Therefore, subsequent efforts will concentrate on nanowires with diameters around 65 nm. However, the diameter-reduced nanowires lead to severe clustering during the printing process, as recorded in Supporting Video S1 and Figure S6. To enhance the uniformity both within individual sensors and across different batches, magnetic fields are applied to manipulate the arrangement of nanowires. Intriguingly, clustering is mitigated, as the magnetic field is oriented in specific directions. Scanning electron microscopy (SEM) confirms a uniform nanowire distribution over a large area (Figure 1b). Notably, the network demonstrates directional alignment along the parallel field component, as further evidenced by anisotropic hysteresis loops (Figure S7). The alignment improves transparency due to reduced light scattering and absorption (Figure 1k). The intertwined network caused by localized entanglement demonstrates exceptional flexibility (Figure 1l), maintaining adhesion to the substrate and preserving continuous percolation pathways even under extreme bending with a curvature radius as small as about 110 μm (Figure 1c), which is among the best reported values (Supporting Table S1). Further, prestraining the hosting substrate along the alignment direction, followed by releasing after the printing process, can introduce arcs or kink-shaped structures along the nanowire length (Figure 1e).^62^ These wavy patterns, combined with mechanical entanglement, could enable remarkable stretchability for the printed sensor, as fully proved in Figure 2f.

**Figure 2:**
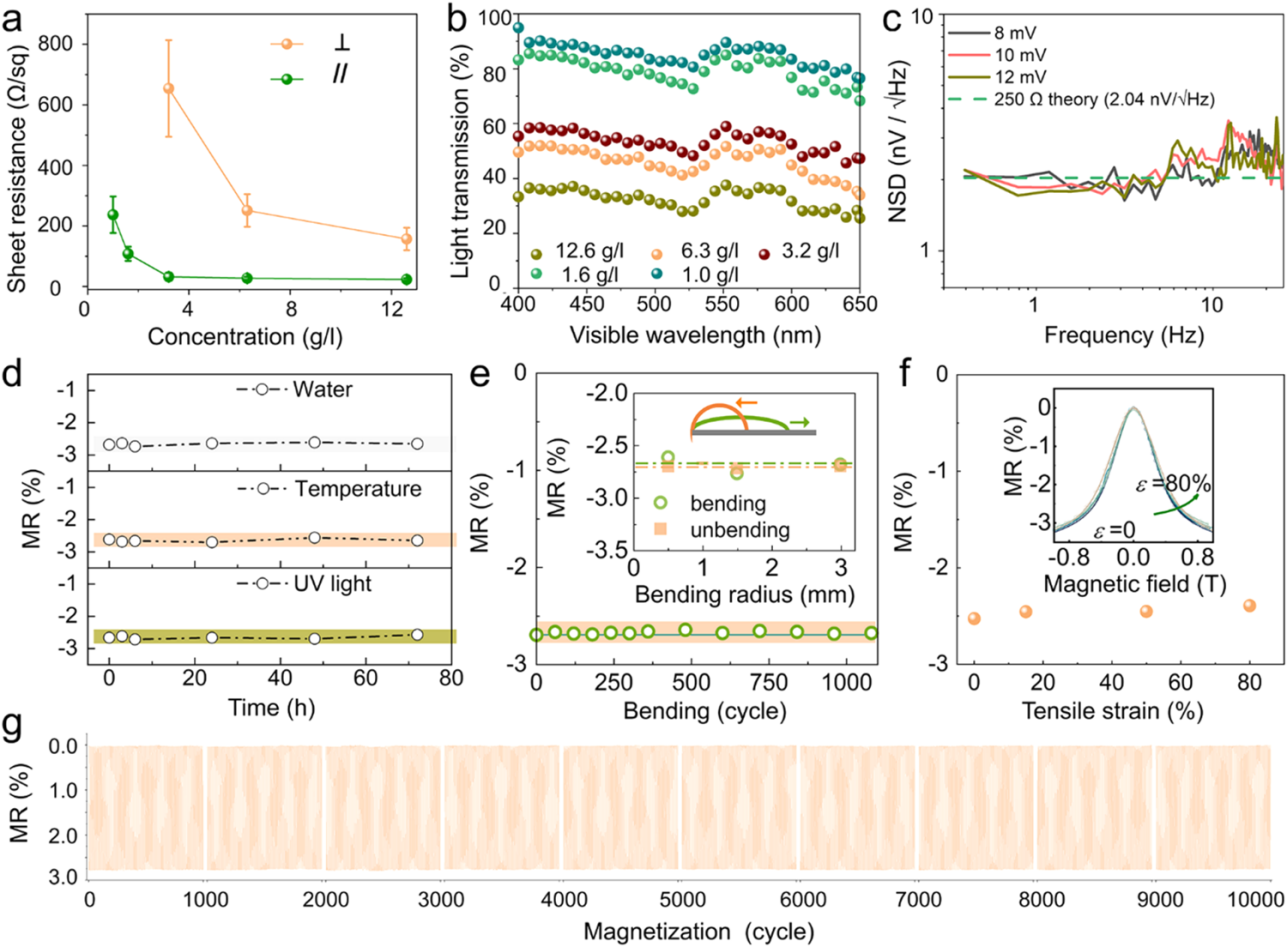
Characterization of the printed magnetoresistive sensors. Evaluation of (a) electrical resistance and (b) optical transparency of the networks printed with the inks of different nanowire concentrations. Electrical conductivities are measured along the alignment direction and the perpendicular direction. Five different concentrations are utilized for the preparation of printable inks, corresponding to approximately 30.7, 15.4, 7.8, 3.9, and 2.4 × 10^11^ pieces of 140-μm-long NiFe nanowires per liter. During ultrasonic treatment to disperse the nanowires in the ink, the nanowires may fracture into several segments, with lengths ranging from a few micrometers to tens of micrometers. (c) Noise spectral density (NSD) of the printed sensor. (d) Magnetoresistance (MR) variation against environmental stressor: water, high temperature (50 °C), and UV illumination. (e) Magnetoresistance (MR) variation as subjected to cycled bending and unbending. Inset: Magnetoresistance ratios are a function of bending radius. (f) Magnetoresistance (MR) variation of the printed stretchable sensor in response to tensile stain. Inset: Magnetoresistance curves were obtained at different strain levels. (g) Magnetoresistance of the printed sensor during 10,000 magnetization cycles. Due to the large volume of data, any 100 cycles are selectively sampled from every 1000 cycles for presentation.

The printed sensor exhibits a maximum magnetoresistance of about 3.5% at around 800 mT (Figure 1i), which is comparable to the saturation ratio (3.6%) observed in the film control (Figure S8). This signals an effective establishment of physical contact between nanowires, predominantly attributed to their unique entanglement configuration. This magnetoresistance ratio is comparable to that of film-based sensors obtained by magnetron sputtering (typically 3%)^26,63^ and falls within the <5% range of electroplated films.^64^ Notably, our nanowire-based sensors exhibit a much higher saturation field (around 600 mT) compared to just a few mT for sputtered films and tens of mT for electrodeposited films. This phenomenon has been attributed to nonuniform demagnetizing field distributed across the lateral cross section of the nanowire, arising from spatial variation in dipolar fields near the nanowire edge.^65^ The aligned sensors demonstrate higher magnetoresistance ratios than those with randomly arranged nanowires (Figure S9), highlighting the importance of spatial alignment. Notably, the magnetoresistance curve exhibits reversal behavior (Figures 1i and S10), which is desirable for sensor applications. Such a trend aligns well with earlier findings,^66^ implying that magnetization rotation dominates over domain wall motion in governing the magnetoresistance response. It is worth noting that the directional alignment results in an exceptional out-of-plane magnetoresistance response, exemplified by a saturation value of 3.5%, which surpasses the in-plane value of 1.3% (Figure 1j). In stark contrast, the thin-film counterpart exhibits the opposite trend, with a stronger in-plane magnetoresistance response but limited sensitivity to vertical magnetic fields (Figure 1j).^67^ The combination of the out-of-plane response (Figure 1j), transparency (Figure 1k), and conformability (Figure 1l) highlights application potential. For example, these features can enhance the touchless experience of wearable electronics by allowing conformal mounting on the skin while minimizing any impact on aesthetic appearance (Figure 1m).

### Characterization of Printed Conformal and Transparent Magnetoresistive Sensors

For practical applications, the repeatability and stability of the sensor performance are systematically analyzed. Resistance values along the alignment direction are found to be at least an order of magnitude lower than those in the perpendicular direction and remain measurable even with low-concentration inks, where electrical conduction in the perpendicular direction is entirely disrupted (Figure 2a). A typical network features a sheet resistance of about 240 Ω/sq exclusively along the alignment direction (Figure 2a). Intriguingly, lower-density inks have a minimal impact on the magnetoresistance response (Figure S11), as the macroscopic magnetoresistance is essentially an average outcome of the intrinsic properties of individual NiFe nanowires in the printed network. As simulated, the network transparency can be optimized by either reducing the nanowire diameter or lowering the surface coverage (Figure S12). Considering both the chemical stability of individual nanowires and the large-area uniformity of printed networks, we experimentally achieve a transparency exceeding 85% (Figure 2b). Although printed networks made of Au, Ag, and Cu nanowires exhibit higher transparency (>90%) and lower sheet resistances (1–20 Ω/sq),^37,68–72^ these noble metals are significantly more expensive, and critically, lack magnetoresistive properties. Notably, the transparency of our magnetoresistive network is comparable to that of commercially available indium tin oxide (ITO) glass (ranging from 75 to 90%, as summarized in Supporting Table S2), indicating its practicality for applications in transparent electronics. Figure 2c records the noise spectral density, which exhibits a similar white noise level for all different bias voltages and matches with the estimated noise voltage of 250 Ω (2.04 nV/√Hz). The linearity range for the output voltage response is within around ±500 mT (Figure S13). The calculated sensitivity (*S* = d*V*/d*H*) at 8, 10, and 12 mV is about 5.7 × 10^–5^, 7.2 × 10^–5^, and 8.3 × 10^–5^ V/T, respectively. Following the definition of detectivity:^73^ *D* = total voltage noise (*V*_n_)/sensitivity (*S*) [T/√Hz], the printed sensor features better than 30 μT/√Hz at 10 Hz at 8 mV, and 20 μT/√Hz at 10 Hz at 12 mV, respectively. To shield the NiFe nanowires from environmental stressors, a thin layer of protective polymer (e.g., polydimethylsiloxane or poly­(methyl methacrylate)) is coated over the printed traces. This coating effectively blocks direct contact with humidity, sweat, and other contaminants. Experimental results show that the encapsulated sensors retain stable magnetoresistance even after 3 days of exposure to water, temperature fluctuations, or UV light, highlighting their durability in real-world conditions (Figures 2d and S14). The entanglement configuration and the encapsulating layer enable the printed sensor to maintain a stable magnetoresistance even under severe deformation (Figures 2e and S15a). After 1000 bending cycles, the magnetoresistance has minimal alteration (Figures 2e and S15b). Thanks to the wavy structure along the nanowire length, the sensor can gain stretchability up to 80%, with negligible magnetoresistance variation across different strain levels (Figure 2f). Notably, the stretchability can be further enhanced by combining meander patterns or wrinkled substrates that are widely used in stretchable electronics (Supporting Table S1). The robust sensor ensures consistent magnetoresistance performance over 10,000 magnetization cycles, which is pivotal for long-term operation (Figure 2g).

### Mechanism for Achieving Mechanical Conformability and Visual Transparency in Printed Magnetic Networks

To unveil the mechanism underlying the unique properties of the printed sensors, both experiments and simulations are conducted to investigate the role of magnetic guidance during the printing process. Following previous reports, parallel fields are initially applied relative to the printed ink layer. The nanowires of ferromagnetic material suspended in the ink interact with the external magnetic fields, generating a magnetic torque that causes the nanowire to rotate and align with the field direction (Figure 3c_1_). The stray fields of the magnetized nanowires are primarily concentrated at the two ends with weaker field strength in the middle region. Moreover, the magnetic field lines in the central area are mostly parallel, making this region ineffective for attracting the surrounding nanowires. In this context, the attraction between the top and bottom ends of adjacent magnetized nanowires drives the elongation of the percolation path along the field direction (Figure 3c_3_) and the parallel nanowires repel each other in the lateral direction (Figure 3c_4_), aligning with previous experimental observations. In our experiments, although nanowire traces are extended along the field direction, severe bundling occurs laterally (Figure 3a_3_). That is because nanowires with reduced diameters and high aspect ratios are prone to mechanical bending, and these bent nanowires no longer act as simple dipoles. Instead, strong magnetic fields are also present around the curved regions, in addition to being concentrated near the ends (Figure 3c_2_). Consequently, strong magnetic forces along the curved profiles lead to lateral nanowire clustering. Intriguingly, strong magnetic interactions between these nanowires may cause mechanical entanglement, effectively enhancing the mechanical robustness of the printed network against deformation, which is in accordance with our experimental measurements (Figure 1c). When magnetic fields are applied perpendicularly to the ink layer, clustering is obviously mitigated (Figure 3a_1_) compared to scenarios without magnetic fields (Figure S6) or with parallel fields (Figure 3a_3_). This improvement could be attributed to the pinning effect, where external magnetic fields and gravity anchor the nanowires onto the substrate, partially counteracting the magnetic attraction of magnetic dipoles in the lateral direction. Based on these observations, an empirical conclusion is that parallel fields extend electrical pathways, while perpendicular fields alleviate unfavorable clustering. To leverage both effects, we applied oblique magnetic fields are applied. To determine the appropriate tilt angles, magnetic fields with continuously varying directions are tested. A gradual transition of nanowire distribution is detected, evolving from small bundles to continuous networks and eventually forming isolated long traces (Figure 3b). Statistical analysis indicates that tilt angles between 50 and 70° are optimal for creating uniform networks, evidenced by improved signal intensity related to the nanowires (Figure 3d) and reduced light transmission (Figure 3e), both resulting from the spreading of nanowires on the substrate. At these appropriate tilt angles, both the pinning effect and the trace elongation effect remain due to the interplay of dipole–dipole interactions, magnetic anisotropy, field-induced forces, and gravity. In contrast, limited by the ink thickness (on the order of hundreds of micrometers), the tilted traces are shorter than those formed under a parallel field. The smaller nanowire traces generate weaker dipole fields, restricting their influence on nearby nanowires to short-range interactions. This further inhibits the formation of large clusters, contributing to uniform nanowire distribution. Once fully dried, the tilted traces lay down onto the substrate under the influence of gravity. Notably, the length of the tilted traces exceeds their projected length on the substrate. For example, at a tilt angle of 60°, the final dried trace on the substrate is twice the length of its horizontal projection. This characteristic facilitates the formation of percolation pathways between adjacent traces, leading to a continuous network across a large area (Figure 3a_2_). The uniform network exhibits stronger angular dependence in magnetization compared to that outside the optimal range (Figures 3f and S16).

**Figure 3:**
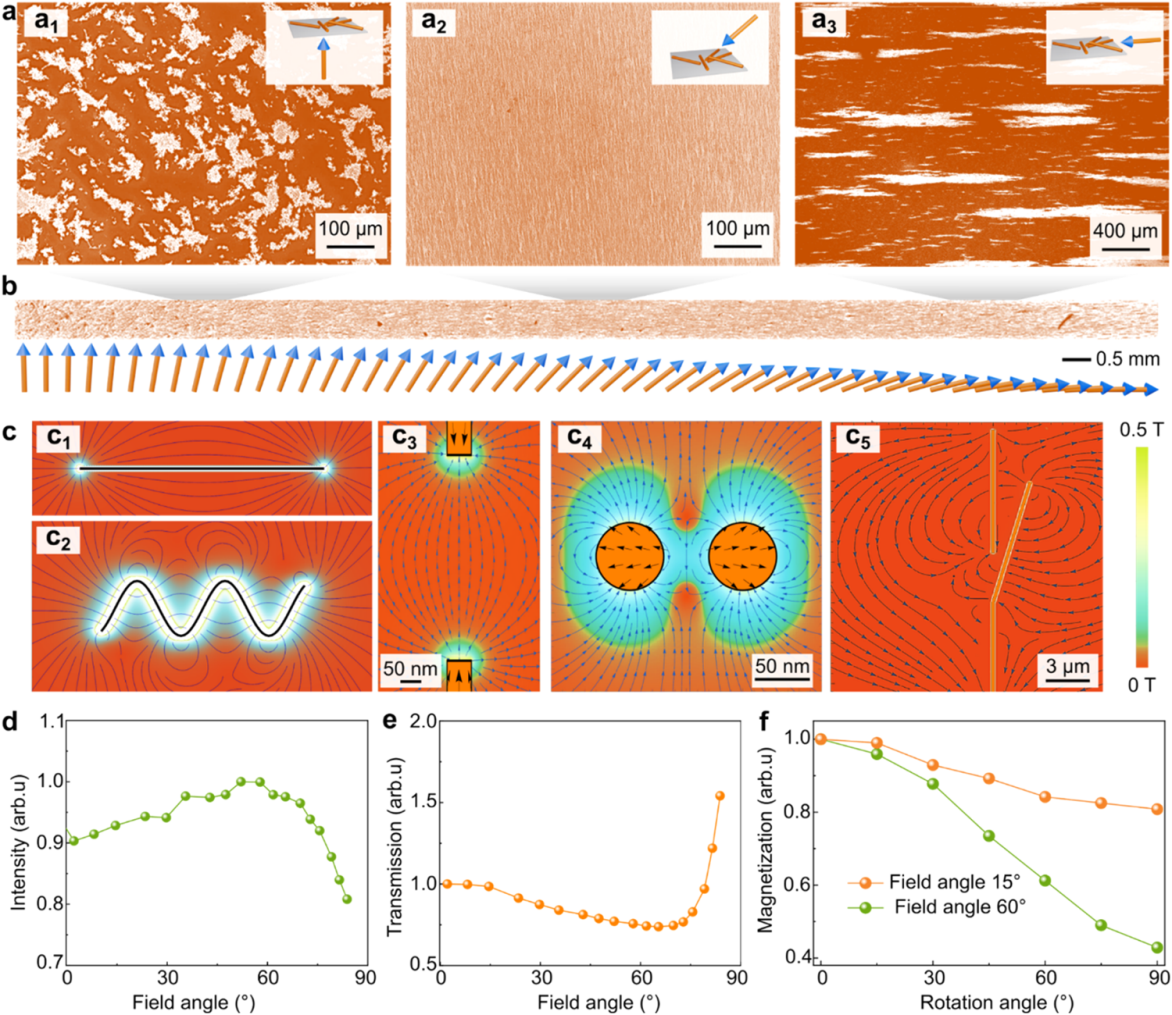
Mechanism of the NiFe nanowire network formation. (a) Nanowire networks printed under the guidance of magnetic fields. The guiding fields are (a_1_) OOP perpendicular, (a_2_) OOP oblique, and (a_3_) IP parallel with respect to the substrate. (b) Nanowire distribution printed in magnetic fields with continuous direction variation. Bottom (from left to right): the orientation of the magnetic field relative to the substrate changes from perpendicular to parallel. Top: a corresponding SEM image of NiFe nanowires printed in the indicated magnetic fields. (c) Magnetic field distribution around magnetic nanowires and the resulting mechanical forces as applying an external magnetic field. We assume that the main nanowires are magnetized along the external field. Cadet blue and black arrows indicate the directions of magnetic fields and mechanical forces, respectively. (c_1,2_) Relative comparison of stray fields around magnetic wires (thick black lines) of (c_1_) straight and (c_2_) sinusoidal profiles. (c_3–5_) Spatial variation of magnetic fields and magnetic forces as two magnetic nanowires are in magnetic fields. In (c_3,4_), two straight nanowires are relatively positioned in different configurations, including (c_3_) the chain-like and (c_4_) parallel arrangements; in (c_5_), one of the nanowires is curved and arranged next to the straight one. The straight nanowires are assumed to be magnetized according to the competition between the Zeeman energy and shape anisotropy. (d, e) Pixel analysis of printed nanowires in (b), including (d) normalized signal intensity of nanowires and (e) normalized light transmission through the network. The signal intensity correlates with the concentration of nanowires distributed over a range of 1 mm slices. As the nanowires are evenly distributed, light transmission is impeded in comparison to scenarios in which nanowires are clustered, leading to reduced transmission with nanowire distribution uniformity. (f) In-plane angle-dependent normalized magnetization of printed networks, printed under magnetic guidance of different angles (i.e., 15 and 60°) relative to the normal direction of the substrate.

### Seamless Integration of Conformal and Transparent Magnetoresistive Sensors into Touchless Interactive Displays

Mechanical conformability and visual transparency, achieved through precise magnetic guidance, allow the printed sensors to seamlessly integrate with various systems for synergistic functionality. As a proof of concept, the sensors can be incorporated into a see-through display system to manage animation without compromising its aesthetic and visual experience. Through leveraging magnetic interaction between the sensor and a magnet-equipped finger, the user’s gestures can be translated into electrical signals. This is achieved by correlating the magnetoresistance variation of the transparent sensor with its relative distance to the magnetic finger (Figure 4a). Thresholds can be defined to trigger various commands (Figure 4b). The high sensitivity allows for the precise detection of subtle variations in gestures, facilitating the execution of fine commands for precise manipulation. Bringing the finger close to the sensor causes a magnetoresistance variation, which is large enough to pass through a preset threshold to enable commands like pausing or resuming a running cat (Figure 4e, Supporting Video S2). As the finger approaches even closer, a substantial increase in the magnetic field around the sensor leads to a notable magnetoresistance rise. Within this range, two thresholds can be established, each serving the purpose of either zooming in or zooming out the animated cat (Figure 4f, Supporting Video S3). Because of the scalable nature and extensive compatibility of the printing technique assisted by magnetic guidance, the sensors can be readily expanded into a matrix, e.g., comprising 4 × 4 sensors (Figure 4c). Real-time monitoring of magnetoresistance changes in the sensor array allows for gesture recognition, such as detecting a swipe by tracking magnetic fingers (Figure 4d_1→2_). It also supports multipoint operations by identifying two or three magnetic fingers simultaneously (Figure 4d_3,4_). These precise signals can support complex operation, satisfying diverse requirements in practical applications.

**Figure 4:**
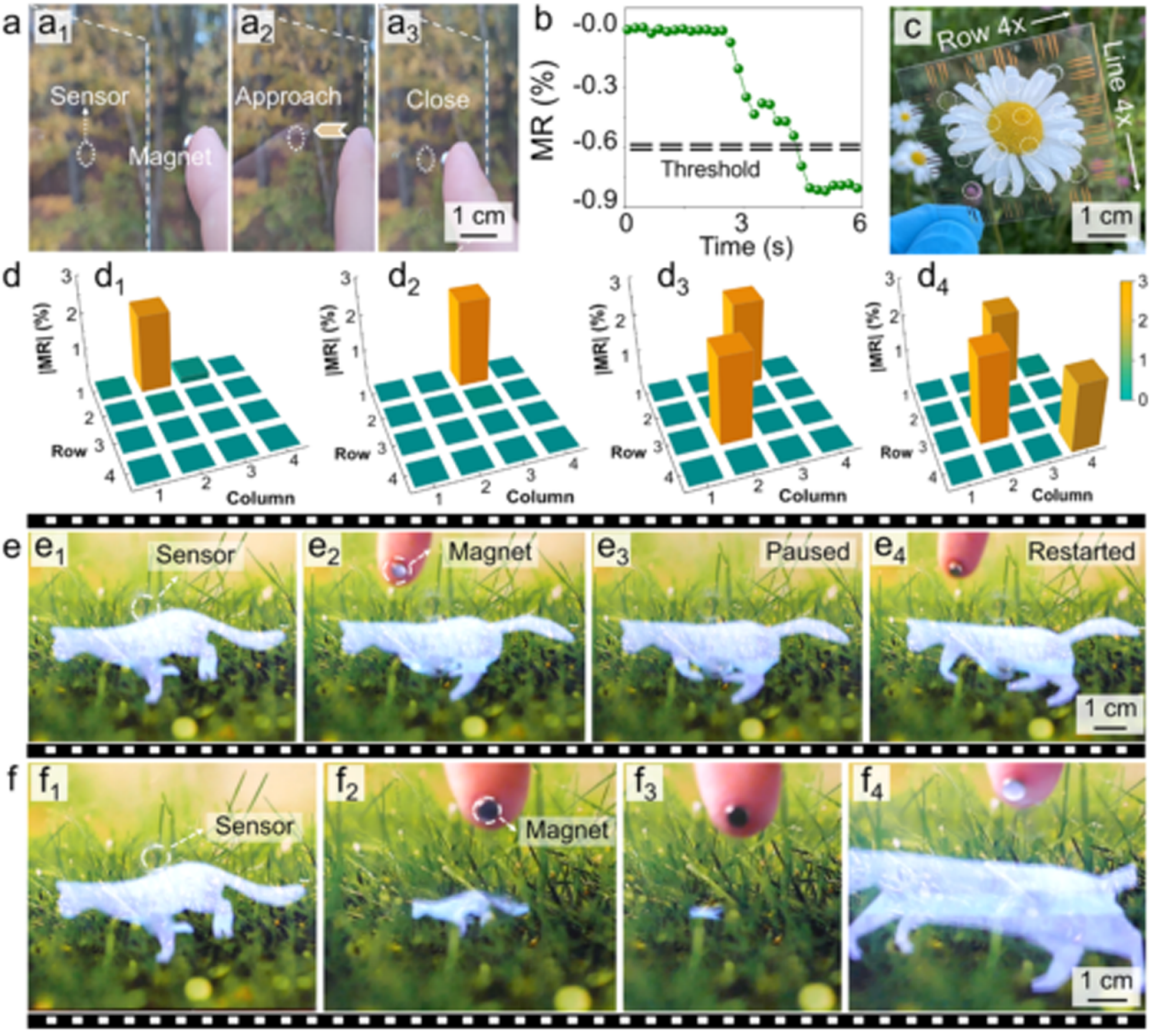
Touchless interaction implemented in see-through displays by integration of printed transparent magnetoresistive sensors. (a) Transparent sensor printed on glass, interacting with a magnet-equipped finger (from left to right, the finger approaches the sensor gradually) and (b) the resulting magnetoresistance (MR) variation. (c) Photograph displaying an array of 4 × 4 magnetoresistive sensors, with each sensor outlined by white dashed lines. Au electrodes are patterned for electrical measurement. (d) Magnetoresistance distribution of a 4 × 4 sensor array for swipe and multipoint interaction. From (d_1_) to (d_2_), a magnetic source moves above the first row of the sensor array. In (d_3_), two and in (d_4_), three magnets are placed over the sensors simultaneously. (e, f) Animation of a running cat, controlled by touchless interaction between the sensor and the magnetic finger. In (e), the running cat can be stopped (from e_1_ to e_3_) or restarted (from e_3_ to e_4_). Please see Supporting Video S2. In (f), the cat can be zoomed out (from f_1_ to f_3_,) or zoomed in (from f_3_ to f_4_). Please refer to Supporting Video S3.

### Environment-Resilient Touchless Interaction in Multifunctional Integrated Systems

The above touchless operation increases interaction safety by eliminating the need for shared surfaces during physical contacts. This feature is crucial for public places requiring high hygiene standards, such as public transport systems with high traffic volumes or hospitals, as schematically represented in Figure 5a. Unlike other touchless counterparts (e.g., optical,^74^ temperature,^75^ or capacitive sensors^76^) magnetoresistive sensors maintain stable functionality even when exposed to environmental interference (Figure 5b), broadening their adaptability. For example, their exceptional resistance to dust and contaminants allows them to function reliably in outdoor and industrial environments, where harsh conditions are common. In contrast, optical and capacitive sensors are susceptible to malfunctions caused by lighting variations or moisture accumulation, whereas magnetoresistive sensors remain unaffected. Furthermore, they can operate through nonmagnetic opaque materials like plastic and textile, making them ideal for concealed or embedded sensing applications. With these advantages, magnetoresistive sensors are poised to advance touchless sensing technology, offering a robust and versatile solution for next-generation applications, e.g., in hospital settings, where blood and other stains are a routine part of medical procedures. As proof of concept, we demonstrate that even when wearing contaminated gloves, magnetic fingers can still control the display of a three-dimensional digital heart (Figure 5c,d). When a magnetic finger approaches the sensor printed on the screen (Figure 5c), its resistance decreases, leading to a voltage change across the sensor (Figure 5d). For example, the voltage of the left sensor drops between 8.5 and 10 s, while the voltage of the right sensor drops between 14.2 and 15.5 s (Figure 5d). These electrical signals can be used to trigger perspective rotation in the *XY* plane (Figure 5c_1_) and the *XZ* plane (Figure 5c_2_), respectively. When two magnetic fingers approach both sensors (Figure 5c_3_), the two voltage signals decrease simultaneously (corresponding to 20–23.5 s in Figure 5d), causing the rotation along an intermediate plane (Figure 5c_3_). This system not only aids in medical diagnosis but also streamlines operation without imposing strict requirements on the operational conditions. The environmental adaptability of the sensors also enables smart windows with reliable interactive functionality (Figure 5e), even as their surfaces are covered with moisture or dust (Figure 5b). By pairing with electronically controlled light filters (Figure S17), the window’s transparency can be dynamically adjusted by sensor manipulation (Figure 5e, Supporting Video S4), we offer effective solutions for optimizing privacy and regulating natural light exposure in smart buildings. Taking this concept a step further, smart windows can be enhanced with energy harvesting capabilities by integrating with transparent thermoelectric generators (TEG), leading to an energy-sustainable system (Figure 5f). With our technique, NiFe nanowires can be printed on designed areas of the smart window, working in tandem with printed Ag nanowires to constitute functional pairs (Figure S18). The transparency tunability allows for the active establishment of localized temperature gradients (as explained in the inset of Figure 5f), which is a crucial prerequisite for efficient thermoelectric conversion. As shown in Figure 5g, the printed thermoelectric generator continuously produces voltage for 3 h under solar illumination, reaching a maximum voltage of approximately 40 μV per unit. In contrast, negligible voltage is generated in the absence of solar illumination, highlighting the effectiveness of the system design. Given the inherent scalability of the printing methods, this system holds high potential for practical applications.

**Figure 5:**
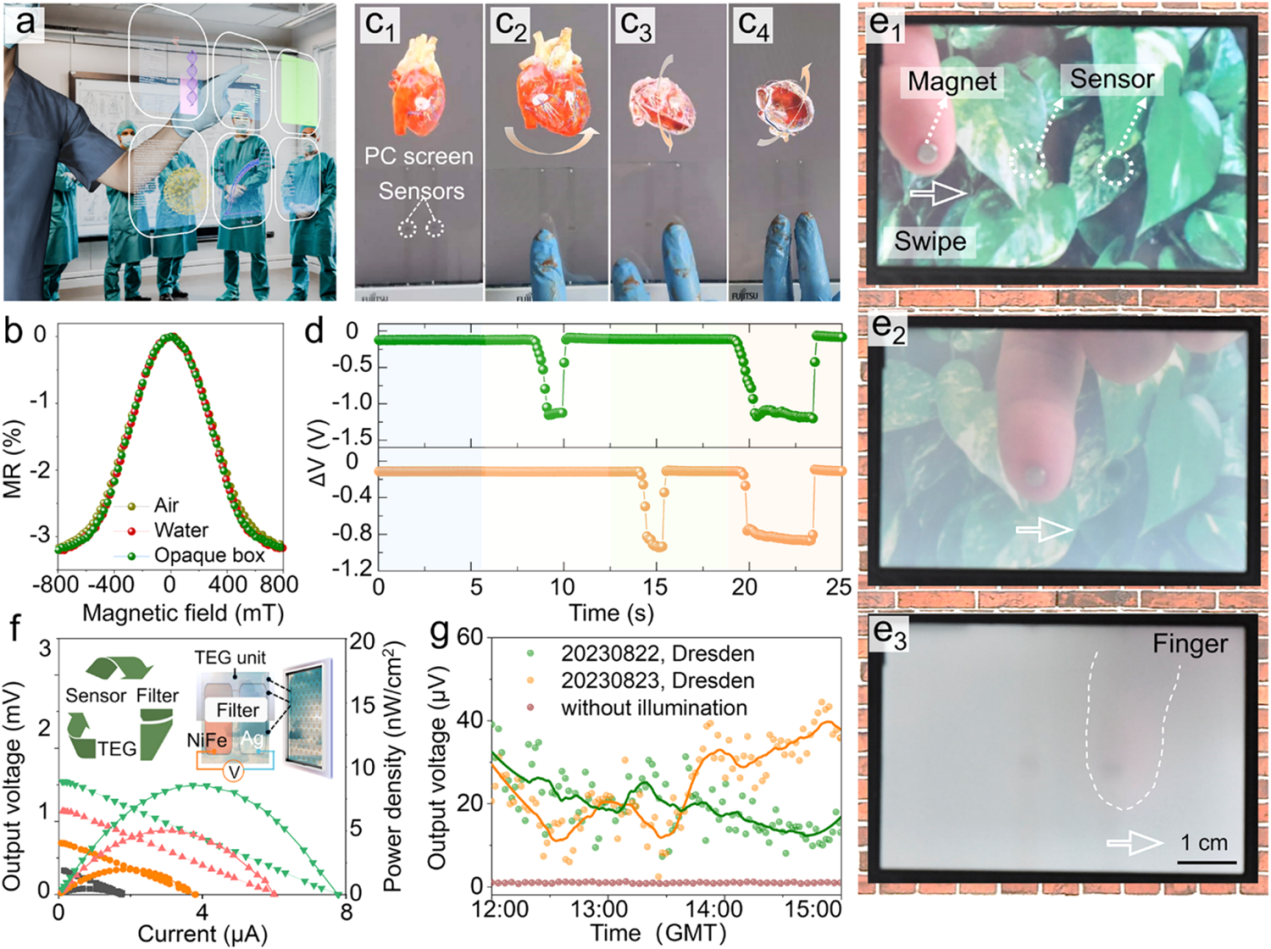
Multifunctional integrated systems incorporating environment-adaptive magnetoresistive sensors. (a) Conceptual illumination of touchless see-through displays in medical application scenarios, enabled by transparent magnetoresistive sensors. (b) Magnetoresistance performance in different operational conditions. (c) Three-dimensional visualization of a virtual heart model displayed on a personal computer, achieving perspective shifts through touchless interactions with two printed magnetoresistive sensors. The sensors work reliably, even if the magnet-decorated gloves are contaminated by liquids and stains. (d) Real-time electrical signal variation of two magnetoresistive sensors during the interaction shown in (c). The top and bottom panels show signals from the left and right sensors, respectively. (e) Smart window with adjustable transparency controlled by magnetoresistive sensors. As a magnetic finger moves across the transparent sensors, the 600 nm light transmission (i.e., transparency) ratios of the electrically controlled light filer (and thus the window) gradually reduce from (e_1_) about 90% to (e_2_) about 50% and (e_3_) less than 10% (Supporting Video S4). (f, g) Smart window with energy conversion capabilities. Inset in (f): Schematics of a solar energy harvesting window through integration of transparent thermoelectric generators (TEG) made of printable NiFe nanowire networks and Ag nanowire networks. Transparency-tunable filters controlled by magnetoresistive sensors are applied to one side of the thermoelectric generator pairs, establishing temperature gradients under solar radiation for thermoelectric conversion. (f) Average output voltage and power density per unit of the printed thermoelectric generator as a function of output current at four temperature differences. (g) Real-time voltage outputs per unit on average and corresponding fitted curves for the printed thermoelectric generator, measured in Dresden, Germany, under varying solar illumination conditions.

## Conclusions

We successfully fabricated conformal and transparent magnetoresistive sensors using deformability-enhanced magnetoresistive nanowires with reduced diameters and high aspect ratios. By engineering magnetic guidance to incorporate both the alignment and pinning effects during the printing process, we overcome the clustering problem of bendable nanowires, achieving large-area aligned nanowires and, thus, enhanced transparency. Moreover, we turn the natural clustering tendency of deformable nanowires to an advantage by utilizing local entanglement to enhance mechanical stability against both bending and stretching. Further, the contactless operation modes enabled by our sensors help to mitigate the spread of bacteria, viruses, and contaminants through shared surfaces, promoting hygienic interactive environments for safer work, living, and entertainment. Their stable performance under harsh conditions enhances their adaptability to practical scenarios, where exposure to dust, liquid, or moisture is common. The environment-resilient touchless interaction capability, coupled with seamless integration potentials, is vital for unlocking the full potential of printed magnetoresistive sensors in rapidly evolving technologies, including the aforementioned customer electronics and smart medicals as well as next-generation immersive education (Supporting Video S5 and Figure S19) and self-sensing transparent soft robotics (Supporting Video S6 and Figure S20). Notably, the developed magnetic guidance technique requires no harsh treatments, harmful chemicals, or even physical contact, underscoring smooth integration into existing manufacturing workflows. This technique is not limited to NiFe nanowires but can be extended to various magnetic nanowires or those with magnetic components with the potential to contribute to a broad spectrum of conformal and transparent printed devices.

However, scaling up this technology for industrial production presents several challenges. These include generating stable magnetic fields across a large-scale printing area, developing a diverse range of magnetically responsive functional inks, ensuring worker safety under prolonged exposure to magnetic fields, and seamlessly integrating the magnetic system into existing printing processes. Achieving this will require advancements in materials science, engineering, equipment design, and workflow optimization, among other areas. It is worth noting that although printed magnetoresistive sensors offer advantages in terms of cost and manufacturing efficiency, their sensitivity is generally lower than that of traditional sensors produced through thin-film processing technology. Consequently, for high-sensitivity applications, further optimization of the sensor design may be necessary. Moreover, the limitations inherent in the printing process make it difficult to fabricate μm-sized sensors, impeding the miniaturization of the integrated systems. Additionally, printed patterns may exhibit unevenness, potentially compromising the stability and consistency of the sensor performance. Therefore, printed magnetoresistance sensors are mainly suited for low-cost, low-precision applications such as simple magnetic field detection, smart packaging, and wearable devices. In high-demand industrial or medical applications, it may be necessary to combine these with traditional sensors or other techniques to overcome the limitations of printed sensors.

## Methods

### Preparation of NiFe Nanowire Inks

NiFe nanowires were prepared by a template-mediated preparation technique, as shown in Figure S1. The anodic aluminum oxide templates used in this project were lab-prepared by anodic anodization of electropolished Al foils. The nanopores within the template feature an average diameter of about 60 nm and a length of about 100 μm. A bilayer of Cr (10 nm)/Au (50 nm) was deposited on the template surface by magnetron sputtering (Ar pressure: 10^–3^ mbar, base pressure: 10^–7^ mbar), serving as conductive electrode in the following electrodeposition. Following this, a layer of Cu (of about 50 μm) was deposited to seal the Al_2_O_3_ pores, playing as the electrode for the electrodeposition of NiFe nanowires and meanwhile preventing the template from cracking in the subsequent steps. Afterward, the unanodized Al was selectively etched off by a mixture of HCl and CuCl_2_. The template holes were then opened from the bottom side using a diluted phosphoric acid solution (0.5 mol/L) at 30 °C for 60 min. The prepared template was immersed in the electrolytic solution of NiCl_2_ (0.4 mol/L), FeCl_2_ (0.01 mol/L), and H_3_BO_3_ (0.1 mol/L) for electrodepositing NiFe nanowires. The pH value of the solution was maintained at 2.5–3.0, and the growth current was set to 8 mA/cm^2^. Following deposition, the board was immersed in a NaOH solution (1 mol/L) to remove the oxide template. Once the oxide was completely dissolved, the remaining nanowires on the Cu foil were transferred into 2-propanol solutions with different volumes for preparing inks of various nanowire concentrations. Finally, the NiFe nanowires were peeled into the 2-propanol solution by ultrasound treatment, and the Cu substrate was removed from the liquid. To prevent clustering of the NiFe nanowires, it is crucial to keep them suspended in solutions. Once completely dried, the magnetic nanowires cannot be uniformly dispersed again when returned into inks. Additionally, avoid using magnets to aid in the collection of NiFe nanowires. After magnetization, these nanowires are prone to aggregation due to magnetic attraction.

### Fabrication of Transparent Magnetic Sensors Printed with NiFe Nanowire Inks

Diverse substrates including polyethylene terephthalate (PET) with 125 μm thickness, Mylar foil with 3 μm thickness, and glass were used for the fabrication of transparent sensors. The conductive pads, used for standard four-point resistance measurement, were constructed from either a 100 nm thick ITO film or a bilayer consisting of a 10 nm thick Cr film and a 100 nm thick Au film. Prior to printing, the ink was shaken for 30 s with a digital vortex mixer (VWR) at a speed of 2500 rpm. Surfactants are not utilized to facilitate dispersion of the NiFe nanowires in the inks because residual surfactants on the surface of the NiFe nanowires after ink drying could potentially hinder electrical conduction between the nanowires. Then, the ink with uniformly dispersed NiFe nanowires was printed on four electrodes. A tilted magnetic field with parallel and vertical components of about 100 and 250 mT, respectively, was applied continuously to the substrate until the printed inks were completely dried. After printing, it is necessary to remove the insulating oxide on the surface of the NiFe nanowires with diluted phosphoric acid (e.g., 0.5 mol/L) for about 30 s in order to ensure the contact quality between the NiFe nanowires for successful electrical percolation. Finally, the printed sensors were coated with poly­(methyl methacrylate) or alternative polymers (e.g., PDMS), followed by solidification at room temperature to prevent possible oxidation.

### Fabrication of Stretchable Magnetoresistive Sensors Printed with NiFe Nanowire Inks

Commercial 3 M VHB tape was initially prestretched with a strain of 150%, after which the ink with uniformly dispersed NiFe nanowires was printed onto it. Subsequent steps, including ink drying and acid treatment, were carried out as previously described. The prestrain applied to the VHB tape was released, allowing the tape to return to its initial strain-free state. Finally, the electrical resistances and the magnetoresistance of the stretchable magnetoresistive sensor under various strains were assessed by using custom-made laboratory equipment.

### Fabrication of Transparent Electrodes

To fabricate sensors shown in Figure 4c, a 50 × 50 mm^2^ PET substrate was coated with AZ 5214E photoresist (MicroChemicals GmbH, Germany) and dried for 2 min at 393 K. Then, 4 × 4 four-point electrodes were written by a DWL 66 laser writer (Heidelberg Instruments, Germany) to establish the patterns of conductive electrodes for the sensor array. In order to ensure high transparency, the width of each patterned wire was reduced to 10 μm. Subsequently, the substrate was heated on a hot plate at 393 K for 2 min and exposed to UV illumination (proMa 140 017, Germany) for 30 s. Afterward, the substrate was developed in AZ 351B solution (MicroChemicals GmbH, Germany) for 60 s, followed by rinsing in deionized water and drying. This process resulted in the acquisition of a PET substrate with predefined layouts. Afterward, a bilayer of Cr (20 nm)/Au (150 nm) was deposited on the PET substrate by magnetron sputtering, and the resulting board was cleaned in acetone to remove photoresist traces, producing a 4 × 4 transparent electrodes array. In addition, we used commercially available ITO-coated glass to fabricate transparent electrodes. The four-point electrode pattern is defined through dry etching using a shadow mask produced by a Silhouette cutting machine. Due to the cutting resolution limitation (approximately 1 mm in this case), the minimum lateral size of a single sensor is around 7 mm. Taking into account the spacing between sensors, the spatial resolution in Figures 4 and 5 exceeds 1 cm. However, the resolution can be further improved by using a miniaturized shadow mask or by combining lithography and vacuum sputtering techniques to form smaller ITO patterns.

### Magnetoresistive Characterization

The magnetoresistive response of the printed sensors was assessed using an electromagnet capable of generating magnetic fields of up to 1 T. The electrical resistance was tested by four-point electrode measurements with a Tensormeter (HZDR Innovation GmbH, Germany) under a current of about 1 mA. The magnetoresistance (MR) ratio was defined as the magnetic field dependence of the electrical resistance:

$$MR_{H_{\mathrm{ext}}}=\left[R_{H_{\mathrm{ext}}}-R_{0}\right]/R_{0}\times 100\%$$

where *R*_H_ext__ and *R*_0_ are the electrical resistances of the sensor under the external magnetic field of *H*_ext_ and zero field, respectively.

### Mechanical Characterization

The magnetic sensor, printed on a PET substrate, was subjected to bending using curved sample holders with radii of 5, 10, 15, and 50 mm. Subsequently, it was positioned between the pole shoes of an electromagnet to assess the bending curvature’s impact on the magnetoresistive properties and mechanical stability. For the printed stretchable sensors utilizing a VHB substrate, they were affixed to a glass holder. By adjustment of the distortion of the VHB substrate, various tensile strains (10, 20, 50, and 80%) were induced on the stretchable sensor to conduct magnetoresistive measurements. The magnetic field was swept across a range of −1 to 1 T, and the electrical resistances of the samples were evaluated through four-point electrode measurements.

### Noise Characterization

We have performed noise measurements using a low-noise voltage preamplifier (SA1) with a noise level of around 270 pV/√Hz and a gain of 1000, and a data acquisition unit (MLA 3, Intermodulation Products) with 16-bit A/D converters. The measurements were carried out with a 100 Hz fast Fourier transform (FFT) bandwidth. The measurement was performed using alternating current (AC) modulation technique with 1010 Hz frequency within trilayer shielding environment for different bias voltages (0.008, 0.01, and 0.012 V). The magnetoresistive sample had a resistance of around 250 Ω.

### Touchless Operation of Magnetic Sensor Array

The transparent sensor array was covered by a 3 mm thick glass to ascertain the minimum touchless operational distance. Permanent magnets, featuring a maximum field strength of 300 mT and a diameter of 5 mm, were brought close to the sensor array perpendicularly, inducing variations in the magnetic field around the sensors. The electrical resistance of each sensor was continuously measured using a digital multimeter (344461A, Keysight).

### Touchless Controlling of Hologram-like Three-Dimensional Display

A holographic-like interface was prepared using Pepper’s ghost illusion. By printing transparent magnetic field sensors on the transparent screen, it was possible to control the animations displayed on the screen. The animations were rendered using the Blender software and controlled through the signal of the sensor read using a USB DAQ 6211 (National Instruments) via NI LabVIEW software (National Instruments). The programmed responses were commanded using the USER32.DLL libraries.

### Fabrication and Characterization of Transparent Thermoelectric Generator

NiFe and Ag nanowires were used to prepare the n- and p-type thermoelectric units, respectively. Inks of Ag nanowires (115 nm in diameter and 20–50 μm in length) were purchased from Sigma. To enhance the transparency of the printed Ag nanowire networks, the inks were diluted accordingly. For thermoelectric characterization, thermoelectric generators were printed on PET foils. The printed p/n-type segments were confined to an area of about 5 × 5 mm^2^. The printing process of the NiFe nanowire inks followed the aforementioned method, and Ag nanowire inks were printed without additional treatment. Two commercial thermoelectric generators were used to build the temperature difference for thermoelectric characterization of the transparent thermoelectric generators. The temperature difference was tuned by changing the applied current. In order to generate a temperature difference on windows, solar illumination is used as the energy source by introducing a commercial light filter. By selectively reflecting and transmitting solar light from one side of the thermoelectric generator, the light filter imparts a temperature difference between the two sides of the thermoelectric generator. The glass with transparent thermoelectric generators was placed outdoors and received direct normal solar irradiation.

### Magnetic Simulation

To calculate the magnetic field around the nanowire of a given profile, we approximate it by a set of *N* = 100 magnetic moments:

1 $${\mathrm{M⃗}}_{i}=\left\{M,0,0\right\},i=\overset{®}{1,100}$$

The dipolar field created around the wire reads

2 $$\mathrm{B⃗}\left(\mathrm{r⃗}\right)=\frac{\mu_{0}}{4\pi }\sum_{i=0}^{N}[\frac{\Delta {\mathrm{r⃗}}_{i}\left(\mathrm{M⃗}\cdot \Delta {\mathrm{r⃗}}_{i}\right)}{\Delta r_{i}^{5}}-\frac{\mathrm{M⃗}}{\Delta r_{i}^{3}}],\Delta {\mathrm{r⃗}}_{i}=\mathrm{r⃗}-{\mathrm{r⃗}}_{i}$$

where μ_0_ is the vacuum permeability and *r⃗*_*i*_ is the position of *i*th magnetic moment. Its value is close to the strength of stray fields created by a nanowire with a large aspect ratio. Here, we consider two shapes: the straight and sinusoidal shapes with the equidistant placement of magnetic moments. Magnetic fields are measured in units of μ_0_ *m*_0_/(4π*r*_0_^2^), where μ_0_ is the vacuum permeability, *m*_0_ is the linear density of magnetic moment along the wire, and *r*_0_ is the unit distance between moments.

## Supplementary Material















## Data Availability

All of the data supporting the conclusions are available within the article and the Supporting Information. Additional data are available from the corresponding authors upon reasonable request.

## Notes

Synthesis and characterization of the NiFe nanowires, magnetoresistance response of the NiFe film and the nanowire networks, transparency modulation of the nanowire networks, stability assessment of the printed sensors, sensitivity and noise measurement, transparent thermoelectric generators, and transparent soft robots (PDF)
Natural drying of printed inks with NiFe nanowires as functional fillers (Video S1) (MP4)
Animation manipulation using transparent magnetoresistive sensors integrated into transparent displays (Video S2) (MP4)
View zooming operated with transparent magnetoresistive sensors in transparent displays (Video S3) (MP4)
Transparency-tunable window controlled by integrated transparent magnetoresistive sensors (Video S4) (MP4)
Three-dimensional visualization of the Brandenburg Gate for immersive education (Video S5) (MP4)
Magnetic transparent butterfly-shaped soft robot (Video S6) (MP4)

## Notes

We applied a permanent magnet to a finger of a user in several studies reported in this manuscript. This magnet is not an electronic component. Furthermore, in several studies, a sensor is applied to the skin of a user. These studies are done according to the ethical approval #SR-EK-459122024 from the ethics committee at the Technical University of Dresden. For this case, we have a written consent of the user (one volunteer, male, 37 years old) who was wearing this sensor. The current lines are always isolated and are not in touch with the skin. The sensor is not worn on the skin for any extended duration.
