# Supplementary material for: Printed Conformal and Transparent Magnetoresistive Sensors for Seamless Integration and Environment-Resilient Touchless Interaction
Source: ACS Nano. 2025 Jun 5;19(23):21891–903. doi: 10.1021/acsnano.5c07664 (PMC12177943; doi:10.1021/acsnano.5c07664)
Supplement: Supplementary file 1 [file nn5c07664_si_001.pdf]

Supporting Information

Printed Conformal and Transparent Magnetoresistive Sensors for Seamless Integration and Environment-Resilient Touchless Interaction

Authors: *Rui Xu[1]*, Eduardo Sergio Oliveros Mata[1], Fei Cheng[1,2], Oleksandr V. Pylypovskyi[1], Qihao Zhang[1], Proloy Taran Das*[1], *Yevhen Zabila[1], Olha Bezsmertna[1], Jun Yang[3], Xiaotao Wang[1], Sebastian Lehmann[3], Lin Guo[1], René Hübner[1], Fabian Ganss[1], Ran He[3], Rico Illing[1], Kornelius Nielsch[3,4], Denys Makarov[1]**

Affiliations: [1]Institute of Ion Beam Physics and Materials Research, Helmholtz-Zentrum Dresden-Rossendorf e.V., Bautzner Landstrasse 400, Dresden 01328, Germany; [2]Xi'an Rare Metal Materials Institute Co., Ltd., Xi'an 710016, PR China; [3]Institute for Metallic Materials, Leibniz Institute for Solid State and Materials Research, Dresden 01069, Germany; [4]Institute of Materials Science, Technische Universität Dresden, Dresden 01062, Germany

*E-mail: r.xu@hzdr.de, d.makarov@hzdr.de

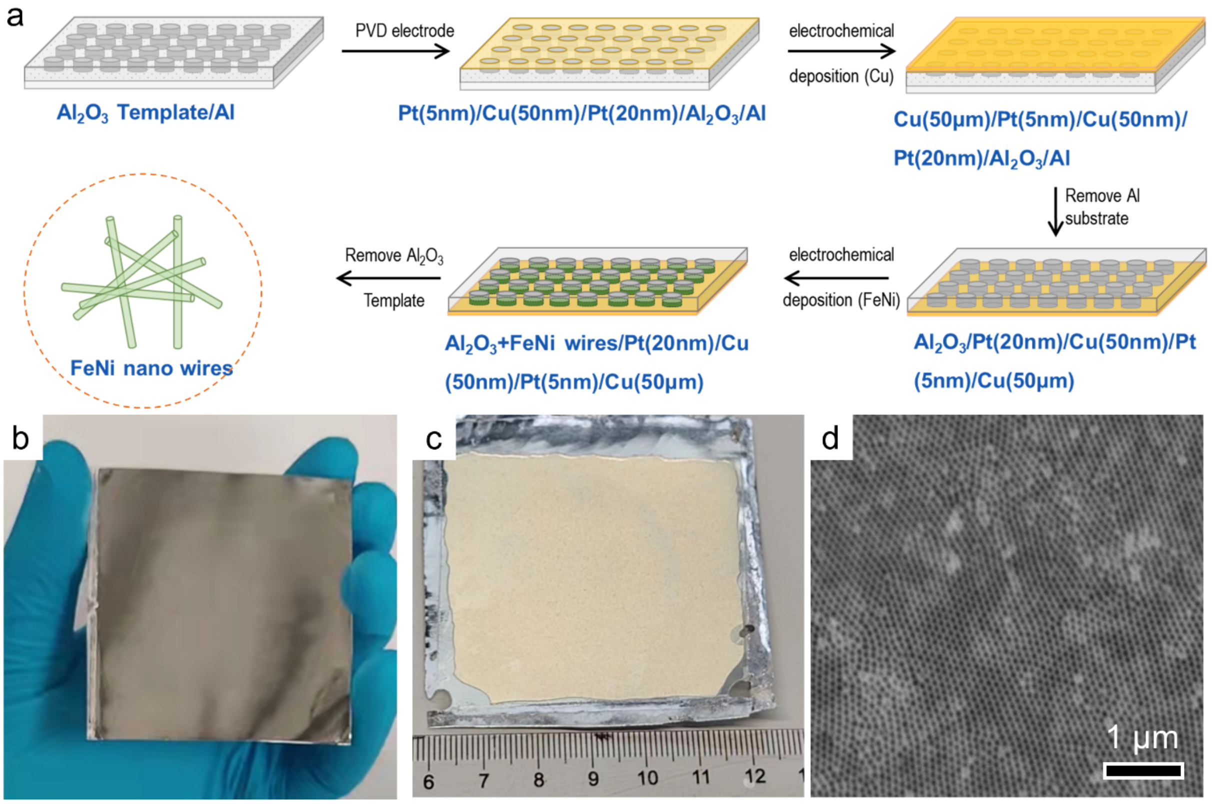

Figure S1. Synthesis of NiFe nanowires. a) Schematic illustration for NiFe-nanowire deposition aided by nanoporous templates. b) Surface-polished Al foil for anodic oxidation. c) Large-area nanoporous template on Al substrate. d) Scanning electron microscope (SEM) image of the nanoporous template.

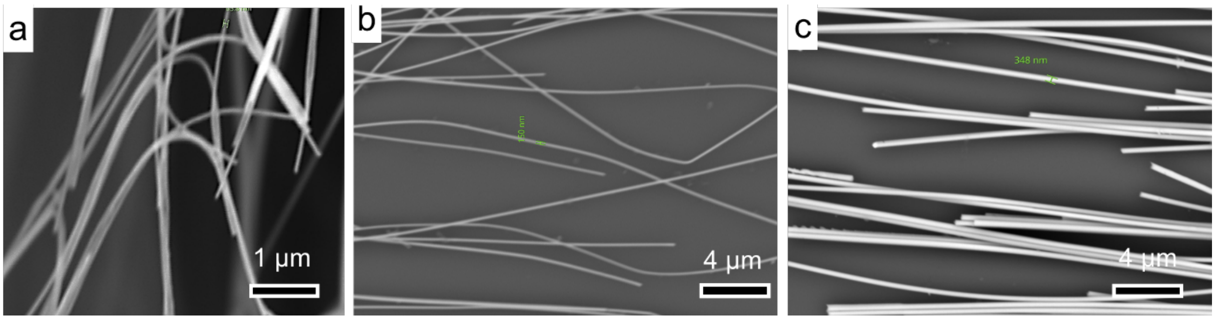

Figure S2. NiFe nanowires of different diameters: about a) 65 nm; b) 150 nm; c) 350 nm.

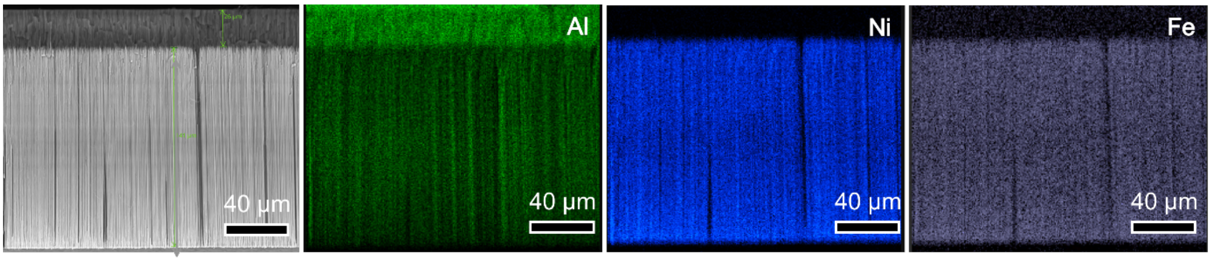

Figure S3. NiFe nanowires filled in nanoporous template and element distribution analysis based on energy-dispersive X-ray spectroscopy (EDXS) in the scanning electron microscope. The nanowires are about 65 nm in diameter and up to 141 μm in length. The length/diameter ratio can be as high as 2170.

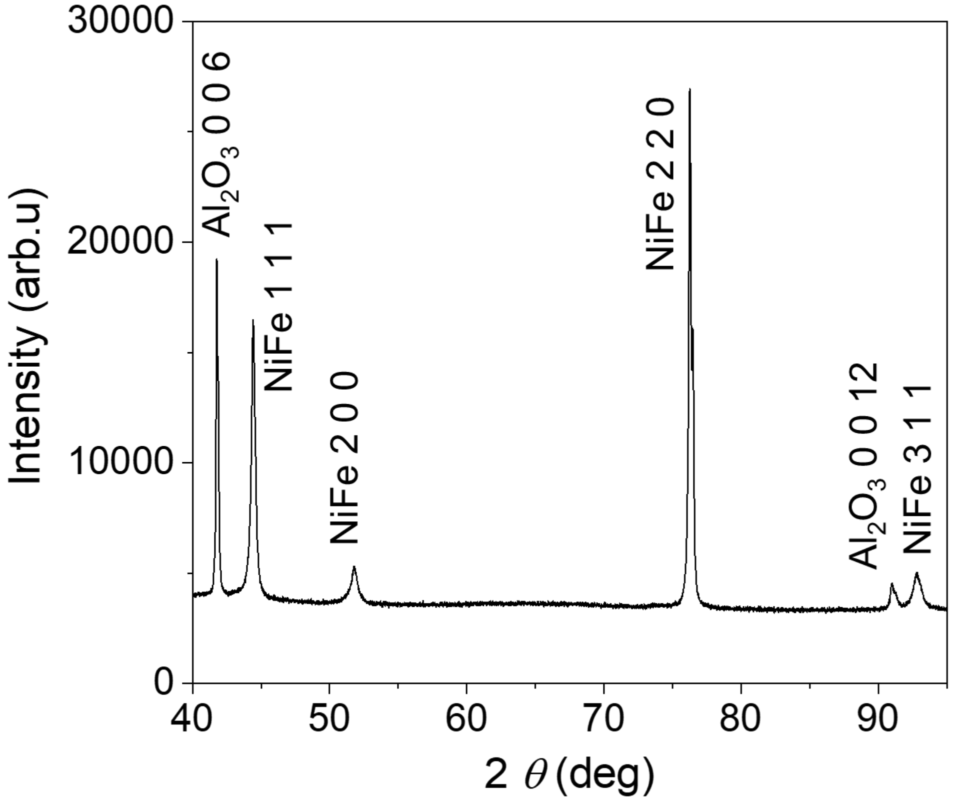

Figure S4. X-ray diffraction (XRD) pattern, revealing four NiFe peaks at 44.5°, 51.8°, 76.4°, and 92.8° corresponding to the {111}, {200}, {220}, and {311} lattice planes, respectively. The remaining Bragg reflections arise from the Al_2O_3 template.

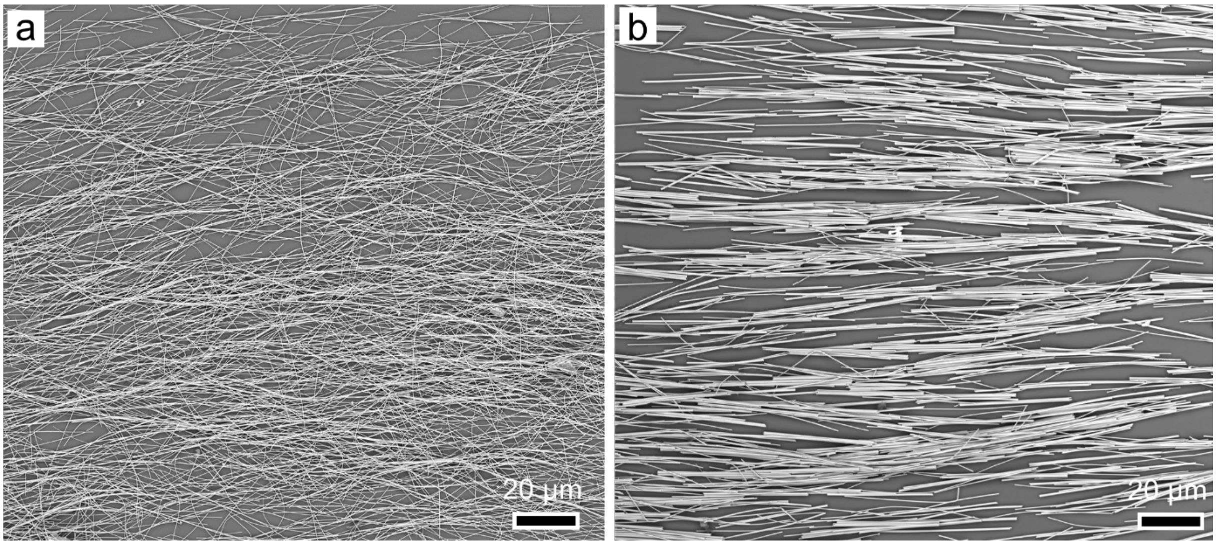

Figure S5. Printed NiFe-nanowire networks with different diameters: a) about 150 nm, b) about 350 nm.

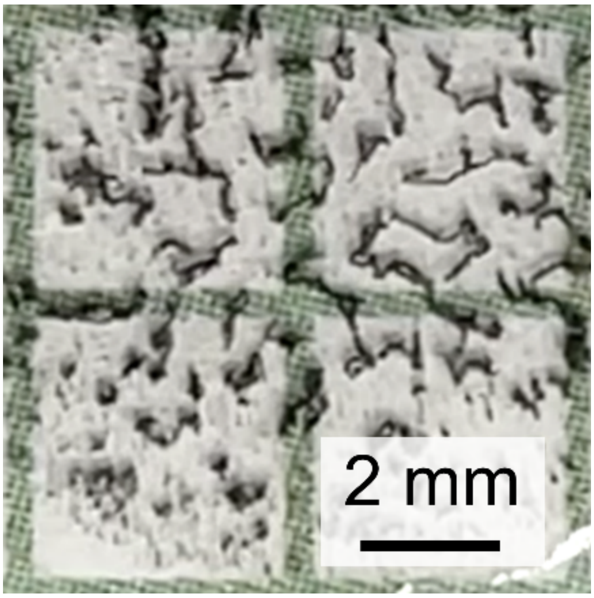

Figure S6. Printed NiFe nanowire network without magnetic field guidance.

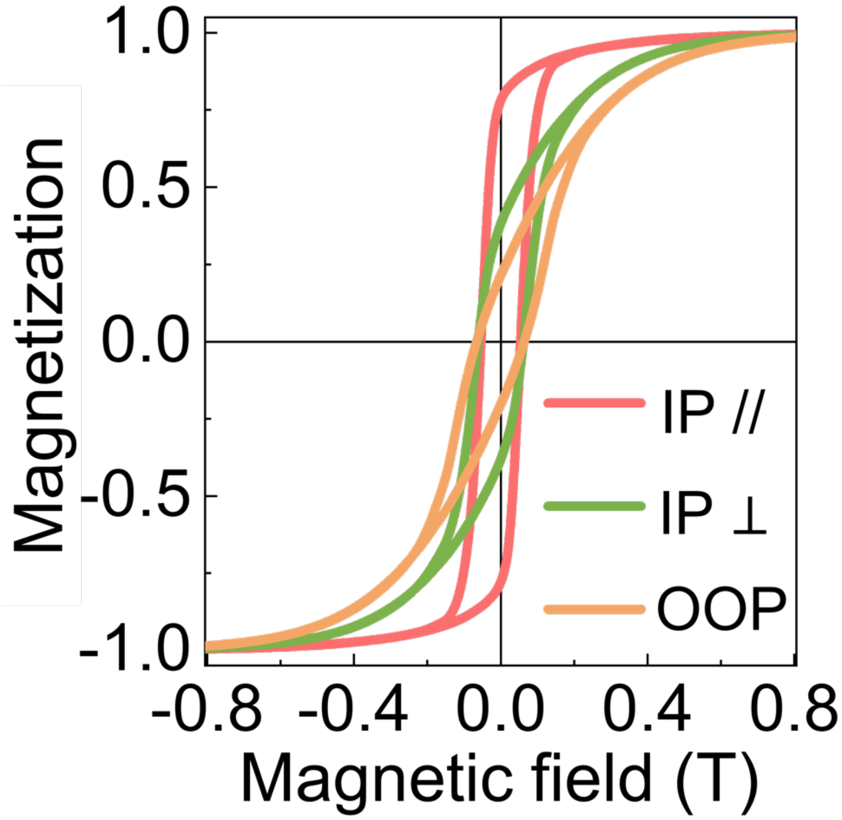

Figure S7. Hysteresis loops of the printed network. Magnetic fields are applied either in-plane (IP) parallel or IP perpendicular or out-of-plane (OOP) perpendicular to the nanowire alignment direction

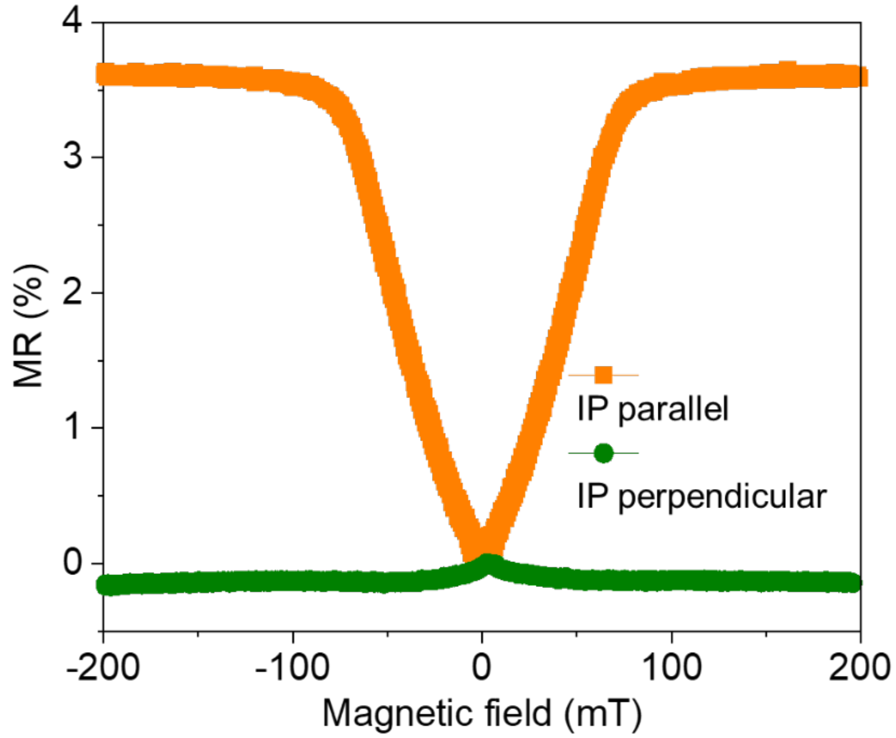

Figure S8. Magnetoresistance (MR) of a magnetic field sensor based on an electrodeposited NiFe film.

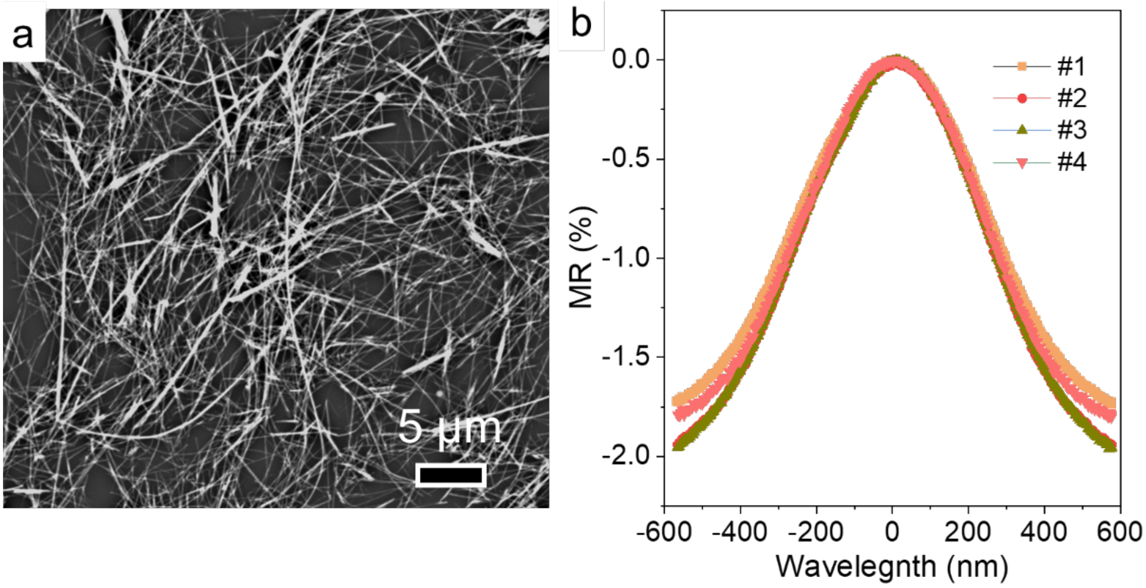

Figure S9. Magnetoresistive networks printed without magnetic field guidance. (a) NiFe nanowires with random distribution. (b) Magnetoresistance response.

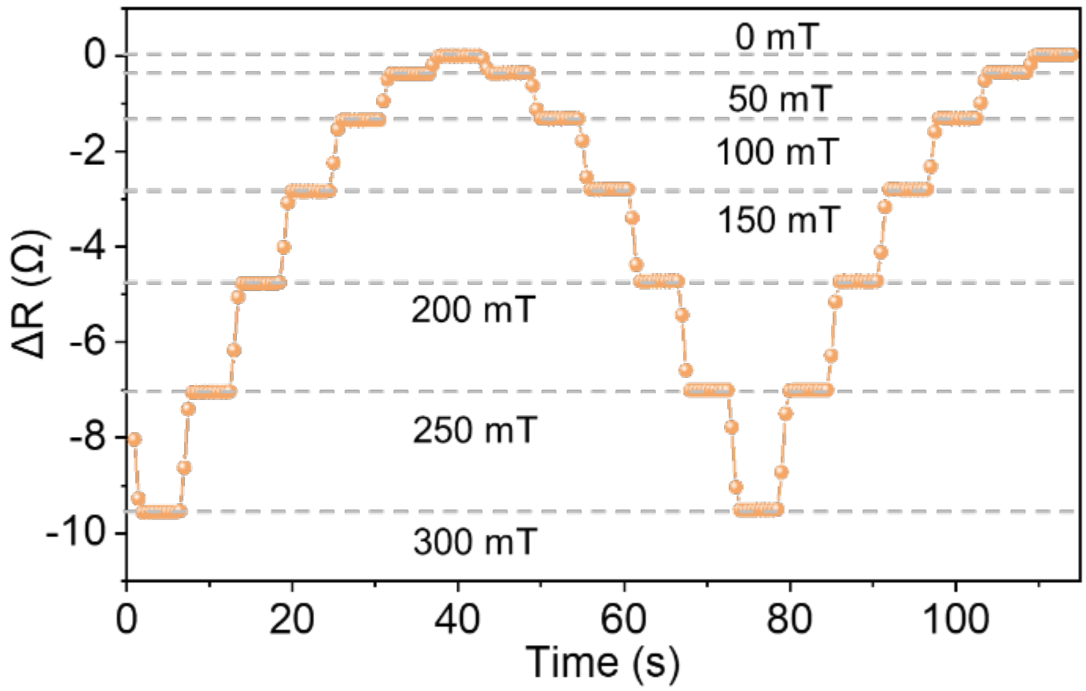

Figure S10. Real-time electrical resistance variation as varying external magnetic fields. The magnetic field decreases from 300 mT to 0 mT, then increases to 300 mT, in steps of 50 mT.

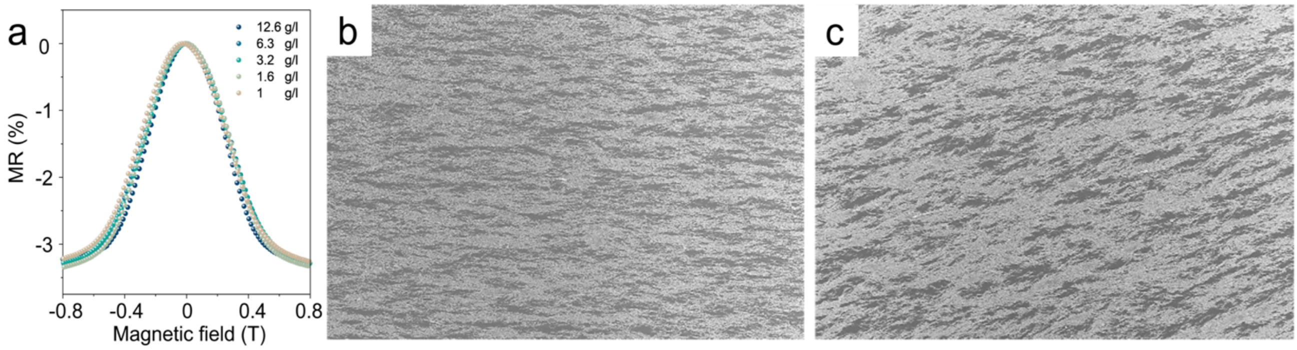

Figure S11. Networks printed with inks of different NiFe nanowire densities. a) Magnetoresistance curves measured for the printed networks. b,c) Networks printed with b) 3.2 g/l and c) 12.6 g/l inks.

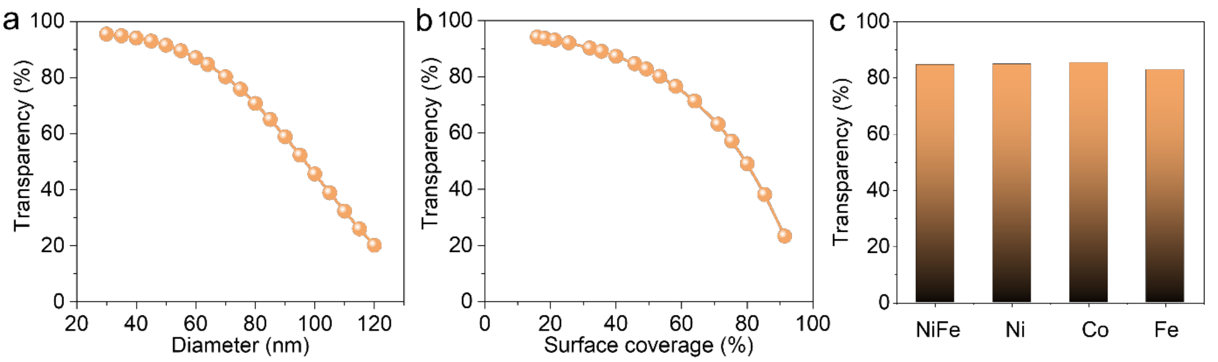

Figure S12. Transparency of magnetic nanowire networks calculated by optical simulation. (a) Transparency as a function of NiFe nanowire diameter. (b) Transparency of NiFe nanowire network with different surface coverage. (c) Transparency of magnetic nanowire network of different materials. Numerical simulations are performed with COMSOL Multiphysics. A plane wave is used to illuminate the NiFe nanowires on a glass substrate. The optical properties of all materials are taken from the built-in parameters of the software. Periodic boundary conditions are applied in the x and y directions.

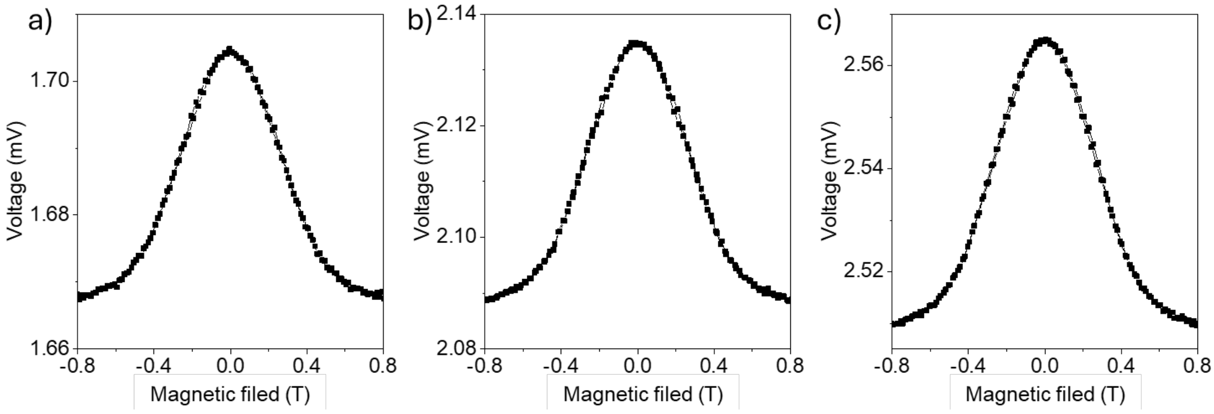

Figure S13. Sensitivity measurements of a printed sensor of NiFe nanowires for different bias voltages: (a) 8 mV, (b) 10 mV, (c) 12 mV. The estimated sensitivity is around 5.7×10^{-5} V/T, 7.2×10^{-5} V/T, 8.3×10^{-5} V/T, respectively.

Sensitivity measurements: We have also carried out sensitivity measurements for different bias voltages[1]. The measurements were performed using Tensormeter measurement device (Tensor Instruments, HZDR Innovation GmbH) and standard electromagnet. We applied the magnetic field perpendicular to the anisotropy axis of the sample in the field range ± 1 T for different bias voltages in between 0.2 mV and 12 mV. The output voltage response with applied magnetic field of the sensor is shown in the Figure S13 and anisotropic magnetoresistance response was found without any hysteresis. The linearity range is found around ± 500 mT. The calculated sensitivity ($S = dV/dH$) at 8 mV, 10 mV and 12 mV is found around 5.7×10^{-5} V/T, 7.2×10^{-5} V/T, 8.3×10^{-5} V/T, respectively.

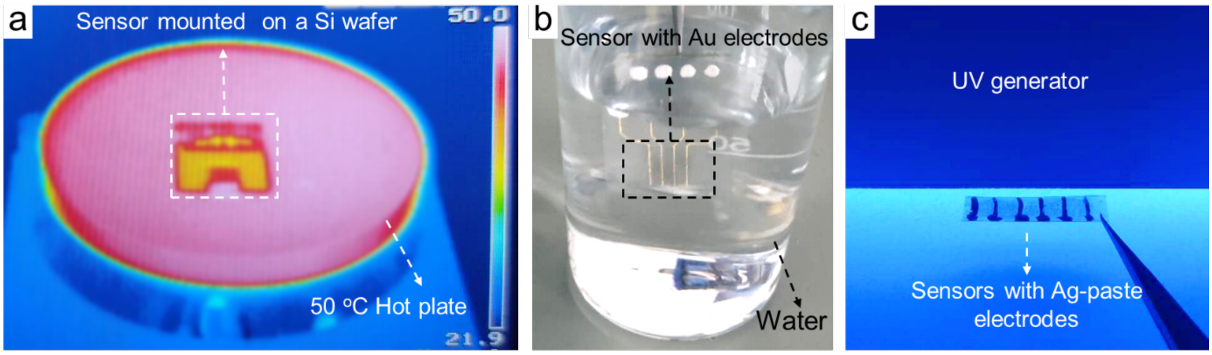

Figure S14. Experimental setup for the sensor stability characterization. (a) Sensor placed on a hot plate heated to 50 °C. (b) Sensor emerged in water. Poly(methyl methacrylate) is coated over the printed trace to protect it from water. (c) Sensor under 365 nm UV radiation.

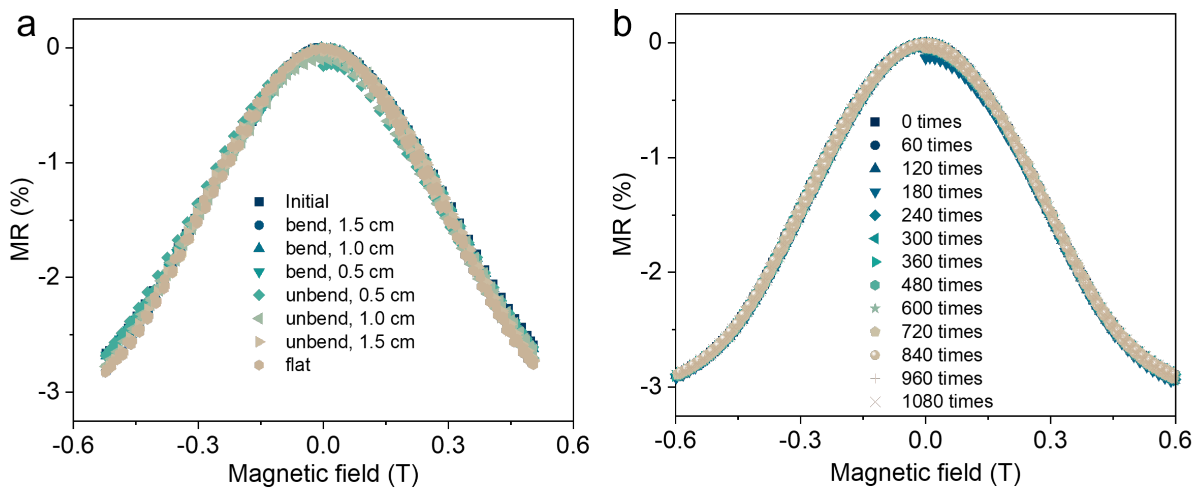

Figure S15. MR performance against mechanical bending/unbending. a) MR variation of the printed nanowire sensor during a bending/unbending cycle with different bending radii (1.5, 1, 0.5 cm). b) MR ratios measured during 1080 cycles of bending/unbending.

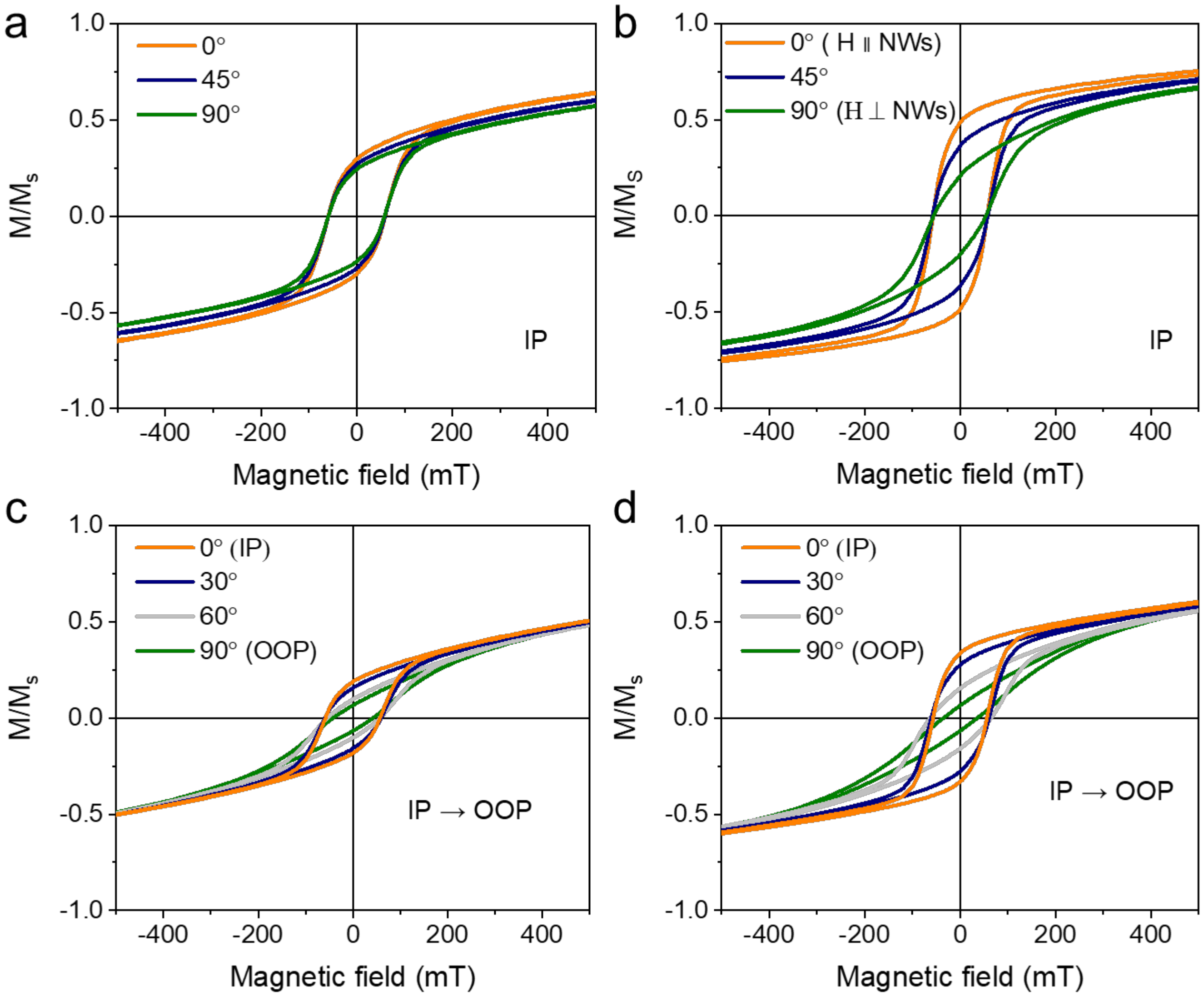

Figure S16. Magnetic hysteresis loops of printed NiFe nanowire networks. The guiding magnetic fields during printing are tilted to the substrate at angles of a,c) 15 and b,d) 60 degrees. In a,b), the magnetic field for hysteresis loops measurement is changed from in-plane (IP) parallel with the alignment direction (denoted as 0°) to in-plane (IP) perpendicular to the alignment direction (denoted as 90°). In c,d), the magnetic field for hysteresis loops measurement is changed from in-plane (IP) parallel with the alignment direction (denoted as 0°) to out-of-plane (OOP) perpendicular to the substrate (denoted as 90°).

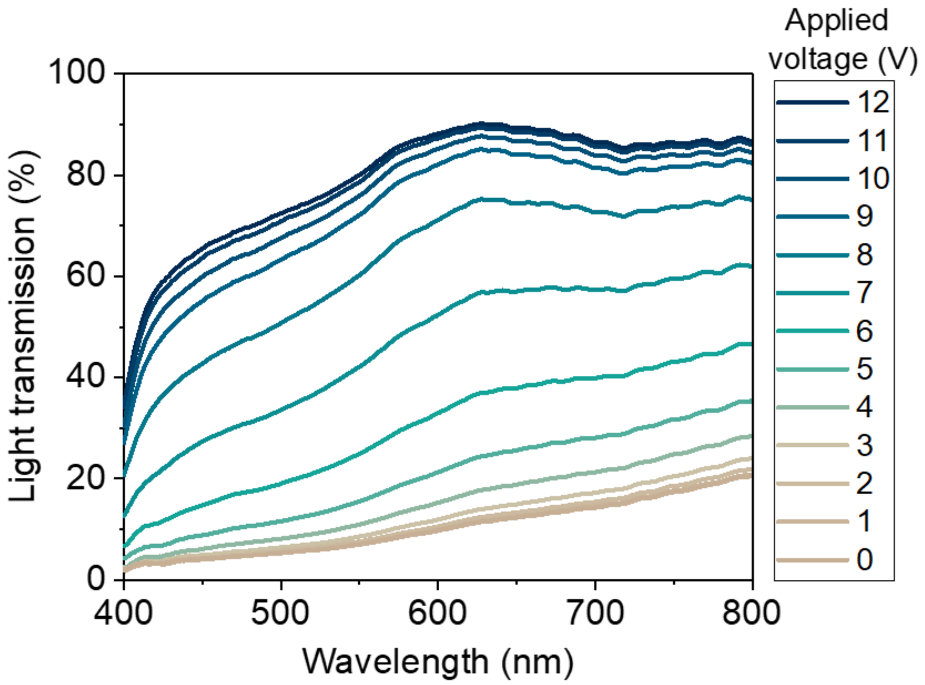

Figure S17. Light transmission spectra of the light filter integrated into smart windows. Its transparency can be adjusted by applying different voltages, e.g., ranging from 0 V to 12 V.

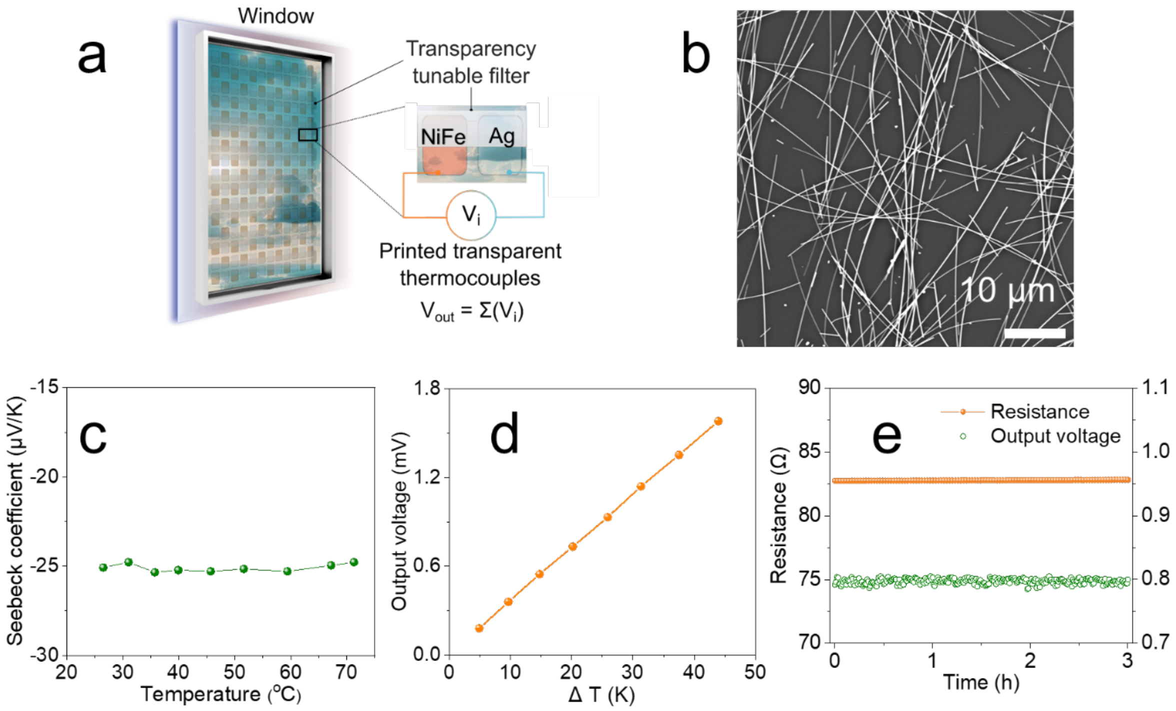

Figure S18. a) Schematic diagram for a window equipped with thermoelectric generators. b) Printed Ag nanowires for functional segments in a thermoelectric generator. c) Seebeck coefficients of the printed NiFe nanowire networks at different temperatures. d) Average output voltage of each unit of the thermocouple generator. e) Stability test for the printed thermoelectric generator.

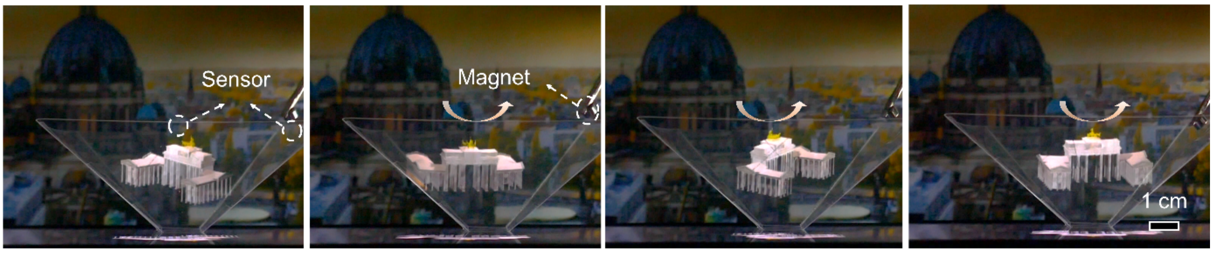

Figure S19. Three-dimensional virtual representation of the Brandenburg Gate, coupled with the observation of perspective shifts through magnetic interactions. Please see Supplementary Movie 5 for details. A virtual 3D Brandenburg Gate can be continuously rotated in 360 degrees by interacting with the transparent sensor, providing users with a unique 3D experience by altering the virtual perspective in real time. This setup can be used in immersive education, for instance, helping students majored in architectures to better experience and understand the details of buildings.

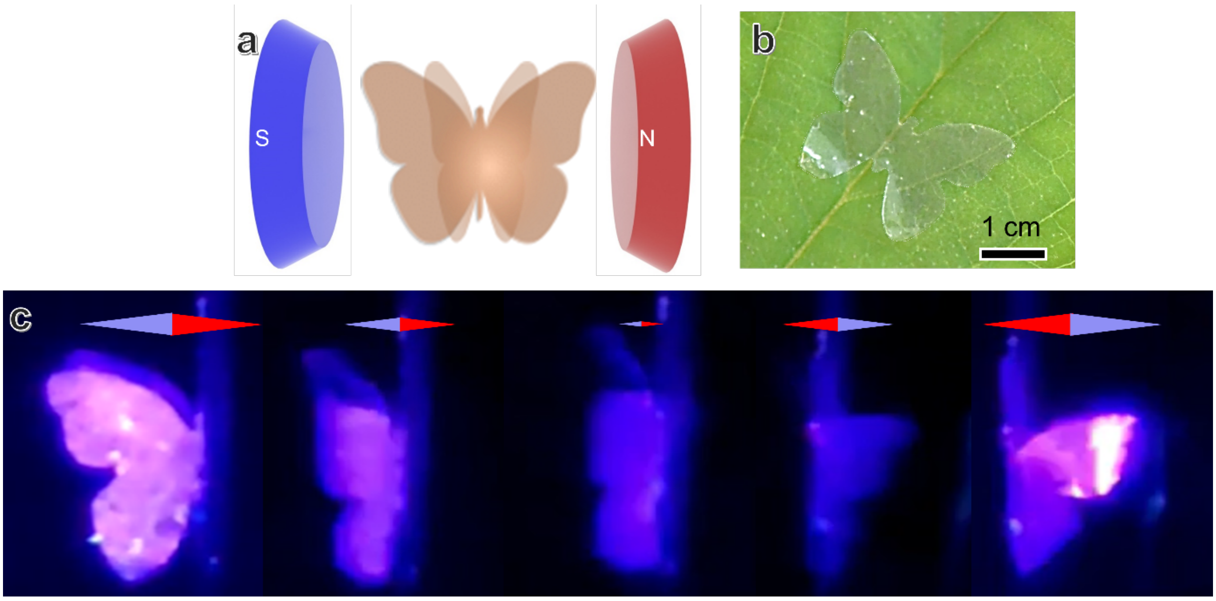

Figure S20. Transparent soft robots. a) Experimental setup for actuating a butterfly-shaped soft robot. b) Transparent butterfly-shaped soft robot made of a spatially aligned nanowire network. c) Butterfly-shaped soft robot controlled by external magnetic fields. To facilitate a clear observation of the transparent robot's movement, UV light renders it in a purple hue. Please refer to Supplementary Movie 6.

The anisotropic magnetic response of flexible networks opens a wide spectrum of applications, e.g., soft magnetic robots (Figure S6). By leveraging both filler-level asymmetry with high aspect ratio and composite-level asymmetry with spatial alignment, the robot features increased magnetic torque with respect to its magnetic material loading, leading to improved actuation capabilities (please refer to Supplementary note 1). Such magnetic actuation allows for remote manipulation without the need of direct contact, and particularly these touchless manipulations are immune to many physical obstacles. This feature renders our robots well-suited for operation in diverse environments, e.g., underwater, confined spaces, or harsh conditions. Notably, the anisotropic magnetoresistance of the printed network (as summarized in Figure 2c) can also be used for detecting the bending angle of the butterfly wings, enabling self-sensing capabilities. In conjunction with the visual transparency that is not easily realizable for traditional magnetic soft robots (Figure S13), our robots enable seamless integration into existing systems without disturbing their original appearance and functionality, further broadening their application possibilities in various fields.

Supplementary note 1

The reason that our robots with aligned NWs feature increased magnetic torque and thus improved actuation capabilities is explained as follows:

The mechanical torque $\vec{T}$ acting on the robot reads

$$\vec{T} = \langle\vec{\mu}\rangle \times \vec{B}$$

where $\langle\vec{\mu}\rangle$ is the average of magnetic moments over the robot's volume and $\vec{B}$ is the external magnetic field. The magnetic moment reads

$$\langle\vec{\mu}\rangle = \frac{1}{V}\int \vec{\mu}\,(\vec{r})d\vec{r}$$

where

$$\mu_x = \mu \sin\theta \cos\phi$$
$$\mu_y = \mu \sin\theta \sin\phi$$
$$\mu_z = \mu \cos\theta$$

angles are functions of coordinate and μ = const. With the preferential ordering along z axis, it is natural to assume that θ angles are distributed uniformly in [0, 2π] range. In this case, the distribution function $f(\theta) = 1/(2\pi)$:

$$\langle\mu_x\rangle = \frac{1}{V}\iint \mu_x\, f(\phi)d\phi d\vec{r} = \frac{1}{2\pi V}\int \mu \sin\theta\, d\vec{r}\int \sin\phi\, d\phi = 0$$

and same for $\langle\mu_y\rangle$. For the z component of magnetic moment, we have

$$\langle\mu_z\rangle = \frac{1}{V}\iint \mu \cos\theta\, g(\theta)d\theta d\vec{r}$$

where $g(\theta)$ is the distribution function by the polar angles of nanowires. Ordering along z means, that $g(\theta)$ is maximal around $\theta = 0$ because of the direction of applied field. This implies $\langle\mu_z\rangle > 0$. Finally, magnetic material loading η influences μ as

$$\mu = \eta M_S V$$

where M_S is the saturation magnetization of permalloy. Thus, the mechanical torque is determined by μ_z and linearly grows with η.

Table S1. Overview of flexible magnetoresistive sensors.

MR type	Functional element	Fabrication process	Structures	Bendability	Stretchability	Transparency	Ref.
GMR	[Co/Cu] multilayer	Sputtering	Wrinkles	Not reported	4.5%	Not reported	[2]
Spin valve	NiFe/CoFe/Cu/ CoFe/NiFe/CoFe/ Cu/CoFe/IrMn	Sputtering	Wrinkles	Not reported	25%	Not reported	[3]
Spin valve	IrMn /CoFe/Cu/CoFe/ NiFe/CoFe /IrMn	Sputtering	wrinkles + ribbons	Not reported	25%	Not reported	[4]
Spin valve	NiFe/CoFe/Cu/CoFe/NiFe/IrMn	Sputtering	Meanders	Not reported	29%	Not reported	[5]
GMR	[Co/Cu] and [NiFe/Cu] multilayer	Sputtering + transfer	Stripes and meanders	Not reported	30%	Not reported	[6]
Spin valve	NiFe/CoFe/Cu/CoFe/NiFe /IrMn	Sputtering	Distributed modulus	Not reported	86%	Not reported	[7]
GMR	[Co/Cu] multilayer	Sputtering	Mesh	250 μm	100%	86%	[8]
GMR	[Co/Cu] multilayer	Sputtering	Pre-strain induced wrinkles	3 μm	270%	Not reported	[9]
Spin valve	NiFe/CoFe/Cu/CoFe/NiFe/IrMn	Sputtering	Film	< 10 μm	Not reported	Not reported	[10]
GMR	[Co/Cu] and [NiFe/Cu]	Printing	Composite	16 μm	100%	Not reported	[11]
AMR	NiFe	Printing	Composite	20 μm	Not reported	Not reported	[12]
AMR	NiFe	Sputtering	Film	100 μm	Not reported	Not reported	[13]
GMR	[NiFe/Cu] multilayer	Sputtering	Film	145 μm	Not reported	Not reported	[14]
AMR	NiFe	Printing	Composite	1 mm	Not reported	Not reported	[15]
Planar Hall effect	NiFe	Sputtering	Film	1 mm	Not reported	Not reported	[16]
Spin valve	[Co/Pd] multilayer	Sputtering	Film	3.5 mm	Not reported	Not reported	[17]
MR	Bi	Printing	Composite	3.5 mm	Not reported	Not reported	[18]
Hall effect	Bi	Sputtering	Film	6 mm	Not reported	Not reported	[19]
AMR	NiFe	Sputtering	Barber pole design	10 mm	Not reported	Not reported	[20]
GMR	[Co/Cu] multilayer	Printing	Composite	10 mm	Not reported	Not reported	[21]
GMR	[Co/Cu] and [NiFe/Cu]	Printing	Composite	10 mm	Not reported	Not reported	[22]
AMR	NiFe nanowire	Printing	Composite	110 μm	80%	85%	This work

MR: magnetoresistance; AMR: anisotropic magnetoresistance; GMR: giant magnetoresistance.

Table S2. Surface resistivity and transmittance of commercial ITO coated substrates.

Ref.	Surface resistivity	Transmittance	Substrate
[23]	70-100 Ω/sq	86%	Glass
[24]	8-12 Ω/sq	84%	Glass
[25]	70-100 Ω/sq	>87%	Glass
[26]	8-12 Ω/sq	>83%	Glass
[27]	15-25 Ω/sq	>78%	Glass
[28]	30-60 Ω/sq	>84%	Glass
[29]	5-15 Ω/sq	>85%	Glass
[30]	60 Ω/sq	>78%	Plastic PET foil

References:

[1] Freitas, P. P.; Ferreira R.; Cardoso S. Spintronic Sensors. *Proceedings of the IEEE* **2016**, *104* (10), 1894-1918.

[2] Melzer, M.; Makarov, D.; Calvimontes, A.; Karnaushenko, D.; Baunack, S.; Kaltofen, R.; Mei, Y.; Schmidt, O. G. Stretchable Magnetoelectronics. *Nano Lett.* **2011**, *11* (6), 2522–2526..

[3] Zou, M.; Bao, X.; Li, X.; Xie, Y.; Yang, H.; Pan, L.; Zhu, X.; Li, R. W. Biaxially-Stretchable Spin Valves with Stable Magnetic Sensing Performance. *IEEE Magn. Lett.* **2024**, *15*, 1–5.

[4] Li, H.; Zhan, Q.; Liu, Y.; Liu, L.; Yang, H.; Zuo, Z.; Shang, T.; Wang, B.; Li, R. W. Stretchable Spin Valve with Stable Magnetic Field Sensitivity by Ribbon-Patterned Periodic Wrinkles. *ACS Nano* **2016**, *10* (4), 4403–4409.

[5] Melzer, M.; Lin, G.; Makarov, D.; Schmidt, O. G. Stretchable Spin Valves on Elastomer Membranes by Predetermined Periodic Fracture and Random Wrinkling. *Adv. Mater.* **2012**, *24* (48), 6468–6472.

[6] Melzer, M.; Karnaushenko, D.; Lin, G.; Baunack, S.; Makarov, D.; Schmidt, O. G. Direct Transfer of Magnetic Sensor Devices to Elastomeric Supports for Stretchable Electronics. *Adv. Mater.* **2015**, *27* (8), 1333–1338.

[7] Pan, L.; Xie, Y.; Yang, H.; Bao, X.; Chen, J.; Zou, M.; Li, R. W. Omnidirectionally Stretchable Spin-Valve Sensor Array with Stable Giant Magnetoresistance Performance. *ACS Nano* **2025**, 19, 5699–5708

[8] Pavlo Makushko, Jin Ge, Gilbert Santiago Cañón Bermúdez, Oleksii Volkov, Yevhen Zabila, Stanislav Avdoshenko, Rico Illing, Leonid Ionov, Martin Kaltenbrunner, Jürgen Fassbender, Rui Xu, D. M. Scalable Magnetoreceptive E-Skin for Energy-Efficient High-Resolution Interaction towards Undisturbed Extended Reality. *Nat. Commun.* **2025**, *16*, 1647.

[9] Melzer, M.; Kaltenbrunner, M.; Makarov, D.; Karnaushenko, D.; Karnaushenko, D.; Sekitani, T.; Someya, T.; Schmidt, O. G. Imperceptible Magnetoelectronics. *Nat. Commun.* **2015**, *6*, 1–8.

[10] Cañón Bermúdez, G. S.; Karnaushenko, D. D.; Karnaushenko, D.; Lebanov, A.; Bischoff, L.; Kaltenbrunner, M.; Fassbender, J.; Schmidt, O. G.; Makarov, D. Magnetosensitive E-Skins with Directional Perception for Augmented Reality. *Sci. Adv.* **2018**, *4* (1), eaao2623.

[11] Ha, M.; Cañón Bermúdez, G. S.; Kosub, T.; Mönch, I.; Zabila, Y.; Oliveros Mata, E. S.; Illing, R.; Wang, Y.; Fassbender, J.; Makarov, D. Printable and Stretchable Giant Magnetoresistive Sensors for Highly Compliant and Skin-Conformal Electronics. *Adv. Mater.* **2021**, *33* (12), 2005521.

[12] Oliveros Mata, E. S.; Cañón Bermúdez, G. S.; Ha, M.; Kosub, T.; Zabila, Y.; Fassbender, J.; Makarov, D. Printable Anisotropic Magnetoresistance Sensors for Highly Compliant Electronics. *Appl. Phys. A Mater. Sci. Process.* **2021**, *127* (4), 1–6.

[13] Cañón Bermúdez, G. S.; Fuchs, H.; Bischoff, L.; Fassbender, J.; Makarov, D. Electronic-Skin Compasses for Geomagnetic Field-Driven Artificial Magnetoreception and Interactive Electronics. *Nat. Electron.* **2018**, *1* (11), 589–595.

[14] Kondo, M.; Melzer, M.; Karnaushenko, D.; Uemura, T.; Yoshimoto, S.; Akiyama, M.; Noda, Y.; Araki, T.; Schmidt, O. G.; Sekitani, T. Imperceptible Magnetic Sensor Matrix System Integrated with Organic Driver and Amplifier Circuits. *Sci. Adv.* **2020**, *6* (4), eaay6094.

[15] Xu, R.; Cañón Bermúdez, G. S.; Pylypovskyi, O. V.; Volkov, O. M.; Oliveros Mata, E. S.; Zabila, Y.; Illing, R.; Makushko, P.; Milkin, P.; Ionov, L.; Fassbender, J.; Makarov, D. Self-Healable Printed Magnetic Field Sensors Using Alternating Magnetic Fields. *Nat. Commun.* **2022**, *13* (1), 6587.

[16] Granell, P. N.; Wang, G.; Cañon Bermudez, G. S.; Kosub, T.; Golmar, F.; Steren, L.; Fassbender,

J.; Makarov, D. Highly Compliant Planar Hall Effect Sensor with Sub 200 nT Sensitivity. *npj Flex. Electron.* **2019**, *3* (1), 3.

[17] Makushko,P.; Oliveros Mata, E. S.; Cañón Bermúdez, G. S.; Hassan, M.; Laureti, S.; Rinaldi, C.; Fagiani, F.; Barucca, G.; Schmidt, N.; Zabila, Y.; Kosub, T.; Illing, R.; Volkov, O.; Vladymyrskyi, I.; Fassbender, J.; Albrecht, M.; Varvaro, G.; Makarov D.; Flexible Magnetoreceptor with Tunable Intrinsic Logic for On-Skin Touchless Human-Machine Interfaces. *Adv. Fun. Mater.* **2021,** *31* (25), 2101089.

[18] Oliveros-Mata, E. S.; Voigt, C.; Cañón Bermúdez, G. S.; Zabila, Y.; Valdez-Garduño, N. M.; Fritsch, M.; Mosch, S.; Kusnezoff, M.; Fassbender, J.; Vinnichenko, M.; Makarov, D. Dispenser Printed Bismuth-Based Magnetic Field Sensors with Non-Saturating Large Magnetoresistance for Touchless Interactive Surfaces. *Adv. Mater. Technol.* **2022**, *7* (10), 2200227.

[19] Melzer, M.; Mönch, J. I.; Makarov, D.; Zabila, Y.; Bermúdez, G. S. C.; Karnaushenko, D.; Baunack, S.; Bahr, F.; Yan, C.; Kaltenbrunner, M.; Schmidt, O. G. Wearable Magnetic Field Sensors for Flexible Electronics. *Adv. Mater.* **2015**, *27* (7), 1274–1280.

[20] Wang, Z.; Wang, X.; Li, M.; Gao, Y.; Hu, Z.; Nan, T.; Liang, X.; Chen, H.; Yang, J.; Cash, S.; Sun, N. X. Highly Sensitive Flexible Magnetic Sensor Based on Anisotropic Magnetoresistance Effect. *Adv. Mater.* **2016**, *28* (42), 9370–9377.

[21] Karnaushenko, D.; Makarov, D.; Stöber, M.; Karnaushenko, D. D.; Baunack, S.; Schmidt, O. G. High-Performance Magnetic Sensorics for Printable and Flexible Electronics. *Adv. Mater.* **2015**, *27* (5), 880–885.

[22] Wang, X.; Guo, L.; Makarov, D. Printed Magnetoresistive Sensors for Recyclable Magnetoelectronics. **2024**, *12*, 24906–24915.

[23] https://www.sigmaaldrich.com/DE/en/product/aldrich/703176

[24] https://www.sigmaaldrich.com/DE/en/product/aldrich/703192

[25] https://www.sigmaaldrich.com/DE/en/product/aldrich/576352

[26] https://www.sigmaaldrich.com/DE/en/product/aldrich/578274

[27] https://www.sigmaaldrich.com/DE/en/product/aldrich/636916

[28] https://www.sigmaaldrich.com/DE/en/product/aldrich/636908

[29] https://www.sigmaaldrich.com/DE/en/product/aldrich/576360

[30] https://www.sigmaaldrich.com/DE/en/product/aldrich/639303
